# Comparison of photo- and thermally initiated polymerization-induced self-assembly: a lack of end group fidelity drives the formation of higher order morphologies†
†Electronic supplementary information (ESI) available: Additional NMR spectra, SEC, TEM and DLS data as well as histograms for the PEG_113_–PHPMA_400_ formulations and MALDI-ToF data for the oligomer study. See DOI: 10.1039/c7py00407a


**DOI:** 10.1039/c7py00407a

**Published:** 2017-04-19

**Authors:** Lewis D. Blackman, Kay E. B. Doncom, Matthew I. Gibson, Rachel K. O'Reilly

**Affiliations:** a Dept of Chemistry , University of Warwick , Gibbet Hill Road , Coventry , CV4 7AL , UK . Email: M.I.Gibson@warwick.ac.uk ; Email: r.k.o-reilly@warwick.ac.uk; b Warwick Medical School , University of Warwick , Gibbet Hill Road , Coventry , CV4 7AL , UK

## Abstract

Polymerization-induced self-assembly (PISA) is an emerging industrially relevant technology, which allows the preparation of defined and predictable polymer self-assemblies with a wide range of morphologies. In recent years, interest has turned to photoinitiated PISA processes, which show markedly accelerated reaction kinetics and milder conditions, thereby making it an attractive alternative to thermally initiated PISA. Herein, we attempt to elucidate the differences between these two initiation methods using isothermally derived phase diagrams of a well-documented poly(ethylene glycol)-*b*-(2-hydroxypropyl methacrylate) (PEG-*b*-HPMA) PISA system. By studying the influence of the intensity of the light source used, as well as an investigation into the thermodynamically favorable morphologies, the factors dictating differences in the obtained morphologies when comparing photo- and thermally initiated PISA were explored. Our findings indicate that differences in a combination of both reaction kinetics and end group fidelity led to the observed discrepencies between the two techniques. We find that the loss of the end group in photoinitiated PISA drives the formation of higher order structures and that a morphological transition from worms to unilamellar vesicles could be induced by extended periods of light and heat irradiation. Our findings demonstrate that PISA of identical block copolymers by the two different initiation methods can lead to structures that are both chemically and morphologically distinct.

## Abstract 2

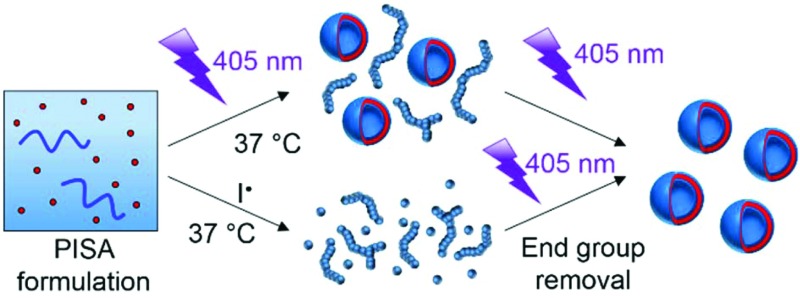
We demonstrate that the PISA of identical block copolymers by either a photo or thermally initiated approach leads to structures that are both chemically and morphologically distinct.

## Introduction

In recent years reversible addition–fragmentation chain-transfer (RAFT) dispersion polymerization-induced self-assembly (PISA) has been shown to be a useful method for obtaining a diverse range of morphologies in aqueous systems,^1^–^8^ alcohols,^9^–^17^ organic solvents^18^–^20^ and ionic liquids.^21^ PISA has several advantages over conventional self-assembly, in that it can be performed at much higher concentrations, it avoids multiple processing and purification steps and morphologies can be reliably targeted after the construction of a phase diagram.^5^,^22^ The reaction parameters known to affect the obtained morphology during PISA reactions include the length and functionality of the solvophilic stabilizer block, the length of the resulting solvophobic block and the concentration at which the polymerization is carried out.^5^,^22^,^23^

Recently there have been several reports of photo-initiated RAFT polymerization being employed in PISA. The first reports of this photoinitiated PISA utilized photoinduced electron transfer catalysts,^24^ or UV light-activated photoinitiators^25^ as a means of radical generation. Tan, Zhang, Sumerlin and co-workers have recently reported the formation of a diverse range of nanostructures in aqueous solution with high conversions in short reaction times using visible light mediated photoinitiated PISA facilitated by sodium phenyl-2,4,6-trimethylbenzoylphosphinate (SPTP) as the photoinitiator. The authors reported using both a brush-like^23^ and linear^26^ poly(ethylene glycol) (PEG) macro chain transfer agent (mCTA) with poly(2-hydroxypropyl methacrylate) (PHPMA) as the core-forming block. In the latter study, the investigators showed it was possible to increase the reaction temperature from room temperature to 50 °C, whilst keeping the reaction kinetics constant. This led to the formation of higher order morphologies, which was attributed to the increased interaction parameter between the core-forming block and the solvent at higher temperatures.^26^ Consequently, such higher order structures were formed in order to minimize interactions between the core-forming block and the solvent.

Boyer and co-workers have demonstrated visible light mediated RAFT PISA in the absence of an external catalyst or initiator to produce a range of morphologies in ethanol/acetonitrile mixtures, using poly(oligo(ethylene glycol)monomethyl ether methacrylate) (pOEGMA) as the stabilizer block and poly(benzyl methacrylate) as the core-forming block.^27^ Two different wavelengths of light were investigated; blue (460 nm) and green (530 nm). Whilst green light irradiation resulted in more complex structures, much slower reaction kinetics were observed under these conditions. Extended reaction times led to 97% conversion and the formation of wormlike micelles with narrow polymer dispersities. In comparison, the use of blue light on an identical polymerization mixture resulted in solely spherical micelles and a much broader polymer dispersity. The authors attributed this difference in morphology to differing degrees of control of the polymerization procedure. The same group also developed a photoelectron transfer RAFT (PET-RAFT) methodology towards photo-PISA using a zinc catalyst, which could be incorporated into the resulting assemblies. The particles showed potential as phototherapeutic agents by virtue of the zinc complex acting as both a photocatalyst for the polymerization and as a singlet oxygen generator.^28^

Zhang and Tan *et al.* utilized photoinitiated PISA to obtain CO_2_-responsive nano-objects in water at room temperature using POEGMA as the hydrophilic corona and a copolymer of 2-hydroxypropyl methacrylate (HPMA) and 2-(dimethylamino)ethyl methacrylate (DMAEMA) as the hydrophobic/responsive core-forming block. The nano-objects underwent dissolution to unimeric chains upon exposure to CO_2_, by virtue of the DMAEMA units becoming protonated in the presence of carbonic acid, which destabilized the cores of the nano-objects.^29^ Similarly, Cai and co-workers used visible light photoinitiated PISA to synthesize nanofilm/silks, ribbon-like, vesicular and tubular structures with tunable pore sizes by introduction of hydrogen bonding and ionic moieties into the core-forming block.^30^ Boyer and coworkers have also developed an oxygen-tolerant photo-PISA system for preparing low volumes of PISA-derived morphologies, which have the potential to be used for the development of self-assembly libraries owing to the lack of the necessity for degassing.^31^ These examples offer simple synthetic routes towards functional materials within minutes of visible light irradiation and demonstrate the power of photoinitiated PISA as a mild synthetic technique towards the preparation of smart self-assemblies with well-defined morphologies. Very recently, Zhang and Tan *et al.* reported a detailed study on the effects of varying the initiation method (*i.e.* light or thermal initiation) and light intensity on the reaction kinetics in the PISA of poly(glycerol methacrylate)-*b*-(2-hydroxypropyl methacrylate) using sodium phenyl-2,4,6-trimethylbenzoylphosphinate (SPTP) as an additional radical source.^32^ However a discussion concerning the morphologies obtained at the various reaction kinetics, or the effect of post-synthetic irradiation was not included.

**Scheme 1:**
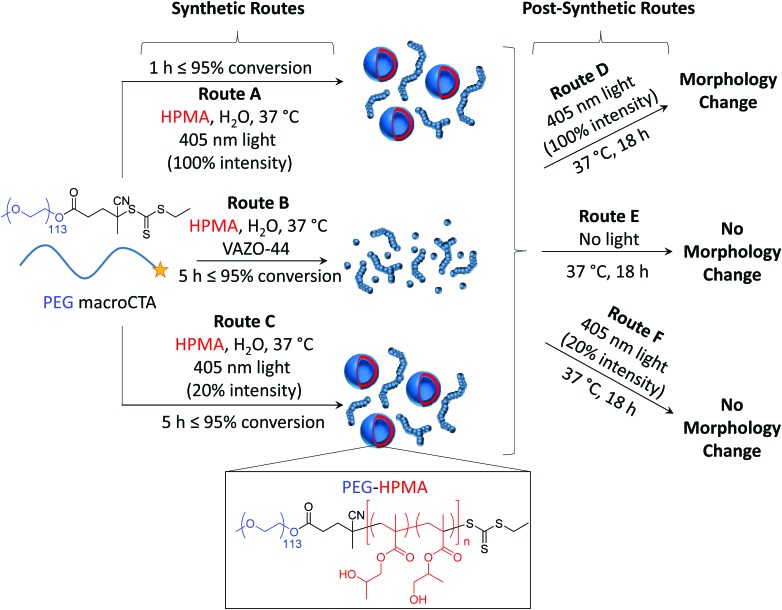
Outline of the synthetic and post-synthetic routes employed in this work. Isothermal photoinitiated PISA at 100% light intensity (route A), thermally initiated PISA (route B) and photoinitiated PISA at 20% light intensity (route C) lead to PEG-HPMA nano-objects. Additionally, irradiation of the pre-formed nano-objects formed by either of routes A, B or C, with 100% light intensity (route D), no light (route E) and irradiation at 20% light intensity (route F) leads to a morphological transition in some instances. The morphology diagrams depict the morphologies of PEG_113_-*b*-HPMA_300_ at 10 wt%, as an example formulation.

As photoinitiated PISA can lead to the formation of well-defined nano-objects within a matter of minutes under mild temperature conditions, it serves as an attractive alternative process to thermally initiated PISA. However, as only a few reports of photoinitiated PISA exist in the literature, it is currently unknown how factors such as the increased reaction kinetics and the presence of a relatively high intensity light source have over the polymerization control, and the control over the morphology of the nano-objects formed. Additionally, since such rich investigation into thermally initiated PISA exists in the literature, it would be of great interest to investigate the ability to predict the final morphology in a photoinitiated process from an existing phase diagram derived from a thermally initiated PISA process. Herein, we attempt to investigate the differences between photoinitiated PISA and conventional thermally initiated PISA. This was achieved by investigating identical phase diagrams formed at the same reaction temperature using both photoinitiated and thermally initiated PISA (Scheme 1, routes A and B, respectively). By investigating the structures formed at 20% light intensity (Scheme 1, route C), the photo- and thermally initiated PISA processes could be studied further under near-identical reaction kinetics. Finally, we also investigated the influence of extended periods of post-synthetic light and heat irradiation (Scheme 1, routes D–F). We found that intense light irradiation of the pre-formed PISA nano-objects for extended periods of time (route D) in the absence of monomer resulted in a morphology change towards higher order morphologies, whereas no light, or low intensity light (routes E and F) did not result in any observed morphology change.

## Experimental

### Methods and materials

#### Materials

The photoinitiator 2-hydroxy-4′-2-(hydroxyethoxy)-2-methylpropiophenone (PP-OH) was purchased from Sigma Aldrich and used as received. The thermal initiator 2,2′-azobis[2-(imidazolin-2-yl)propane]dihydrochloride (VAZO-44) was purchased from Wako Chemicals GmbH and used as received. The monomer 2-hydroxypropyl methacrylate (mixture of isomers, HPMA) was purchased from Alfa Aesar and was passed through a column of basic alumina prior to use. The synthesis of the PEG_113_ mCTA from PEG_113_ monomethyl ether and 4-cyano-4-(((ethylthio)carbonothioyl)thio)pentanoic acid (CEPA) has been described by Tan, Sumerlin and Zhang *et al.* in a previous report.^26^ The MALDI-ToF MS matrix 2′,6′-dihydroxyacetophenone and trifluoroacetic acid sodium salt (NaTFA) were purchased from Sigma Aldrich and used as received. Formvar coated copper grids were purchased from EM Resolutions.

#### Polymer characterization

SEC analyses were performed on a Varian PL-GPC 50 Plus instrument fitted with mixed C columns and RI and UV detectors using 5 mM NH_4_BF_4_ in DMF as the eluent. Molecular weight distributions were calculated using poly(methyl methacrylate) standards. ^1^H NMR spectroscopy was performed at 300 MHz on a Bruker AV-300 spectrometer. Chemical shifts (*δ*) are reported in parts per million (ppm) relative to the residual CH_3_OH solvent peak at 3.31 ppm. MALDI-ToF MS analysis was performed using 2′,6′-dihydroxyacetophenone as the matrix. A small amount of the solid matrix was deposited onto the plate before a 1 : 1 mixture of the polymer (2 mg mL^–1^ in tetrahydrofuran) and NaTFA (10 mg mL^–1^ in acetonitrile) were deposited as a single solution. Distributions were calibrated against broad PEG standards of known molecular weight.

#### Particle characterization

Transmission electron microscopy (TEM) analysis was performed on either a JEOL 2100 FX or a JEOL 2000 FX microscope. Samples were diluted then deposited onto formvar grids. After roughly 1 min, excess sample was blotted from the grid and the grid stained with an aqueous 1 wt% uranyl acetate solution for 1 min prior to blotting, drying and microscopic analysis. Dynamic light scattering (DLS) analysis was performed on either a high-throughput Wyatt DynaPro Plate Reader II or a single channel Malvern Zetasizer Nano S instrument.^33^ Samples were diluted with filtered deionized water (0.45 μm, nylon) and the diluted samples were not filtered prior to analysis so as to ensure larger structures remained in solution.

#### Light source setup

The light source for the photoinitiated PISA reactions (TruOpto OSV5X3CAC1E) was purchased from Rapid Electronics and had an output power of 800 mW at 12 V DC operating at a wavelength of 400–410 nm. This was fitted to a custom-built setup fitted with a dimmer switch for controlling the output light intensity.

### Polymer synthesis

#### Photoinitiated PISA of PEG-*b*-HPMA diblock copolymers (routes A and C)

All photoinitiated reactions were performed in a custom-built photoreactor setup. This ensured the reaction mixture was only exposed to the light from the 400–410 nm LED light source placed underneath the sample. A typical experiment, to achieve PEG_113_-*b*-HPMA_300_ at 10 wt% HPMA was as follows. PEG_113_ mCTA (100 mg, 19 μmol) and HPMA (820 mg, 5.7 mmol) were dissolved in deionized water (7.38 mL) in a sealed 20 mL scintillation vial with a stirrer bar either with or without PP-OH (0.85 mg, 3.8 μmol). The mixture was degassed by sparging with N_2_ for 15 min. The sealed vial was incubated at 37 °C with magnetic stirring under irradiation of the 400–410 nm light source at 100% for 2.5 h (route A) or 20% power for 5.5 h (route C) to ensure full monomer conversion. After this time, the vial was opened to air and allowed to cool to room temperature before conversion ^1^H NMR spectroscopic and SEC analyses. ^1^H NMR of the pure polymer was obtained after lyophilization of an aliquot of particles. TEM and DLS analyses were performed on samples after dilution to an appropriate analysis concentration.

#### Thermally initiated PISA of PEG-*b*-HPMA diblock copolymers (route B)

A typical experiment, to achieve PEG_113_-*b*-HPMA_300_ at 10 wt% HPMA, was as follows. PEG_113_ mCTA (100 mg, 19 μmol) and HPMA (820 mg, 5.7 mmol) were dissolved in deionized water (7.28 mL) in a sealed 20 mL scintillation vial with a stirrer bar. The mixture was degassed by sparging with N_2_ for 15 min. After this time, VAZO-44 (1.2 mg, 3.8 μmol) in deionized water (100 μL) was added and the mixture degassed for a further 10 min. The sealed vial was heated to 37 °C with magnetic stirring overnight to ensure full monomer conversion. After this time, the vial was opened to air and allowed to cool to room temperature before conversion ^1^H NMR spectroscopic and SEC analyses. ^1^H NMR of the pure polymer was obtained after lyophilization of an aliquot of particles. TEM and DLS analysis was performed on samples after dilution to an appropriate analysis concentration. ^1^H NMR (300 MHz, CD_3_OD) *δ*/ppm: 4.75 (br s, OH), 4.00 and 3.85 (br s, CH and CH_2_ of PHPMA side chain), 3.63 (br s, CH_2_CH_2_O of PEG), 3.61 (br s, CH of PHPMA side chain), 3.36 (s, CH_3_ of PEG end group), 2.34–1.70 (br m, CH_2_ of PHPMA backbone), 1.70–0.50 (br m, CH_3_ of PHPMA backbone and CH_3_ of PHPMA side chain).

#### Kinetic study

For the thermally initiated and photoinitiated reactions used in the kinetic study, PEG_113_–PHPMA_300_ at 10 wt% HPMA formulations were carried out as described above in a sealed ampule. DMF (42 mg, 57 μmol) was also added at the start of the reaction as an internal standard. The total integral of the vinyl proton peaks at 6.03 and 5.99 ppm, corresponding to one vinyl proton on each of the two isomers of HPMA, was compared to the integral of the DMF CH proton at time = 0 min. Aliquots were taken at various time points and analyzed by ^1^H NMR spectroscopy. The percentage decrease in the integral corresponding to the vinyl protons normalized to DMF, relative to the integral at time = 0 min was used to calculate the monomer conversion. To ensure the reaction vessels remained degassed as aliquots were taken, the reactions were performed in ampules under a continuous nitrogen flow.

#### Post-synthetic procedures (routes D–F)

In a typical experiment, an aliquot of the pre-synthesized formulation was placed in a 20 mL scintillation vial with a screw-top lid. No degassing of the sample was performed. The sample was incubated at 37 °C with 400–410 nm light irradiation at either 100% (route D), 0% (route E) or 20% (route F) power for 18 h. After this time, the mixture was cooled to room temperature prior to analysis.

#### Synthesis of oligomers for end group analysis study

HPMA (1.00 g, 6.9 mmol), AIBN (6 mg, 35 μmol) and CEPA (91 mg, 3.5 μmol) were dissolved in ethanol (2 mL) and degassed in an ampule by purging with argon for 15 min. The vessel was sealed and the reaction mixture stirred and heated at 65 °C for 7 h. After this time, the solvent was removed and an aliquot was purified and fractionated by preparative SEC (mixed B columns) using dimethyl sulfoxide as the eluent. One fraction of which was used for all further analysis. ^1^H NMR (300 MHz, CD_3_OD) *δ*/ppm: 4.75 (br s, OH), 4.00 and 3.85 (br s, CH and CH_2_ of side chain), 3.61 (br s, CH of side chain), 2.33–1.70 (br m, CH_2_ of backbone), 1.70–0.50 (br m, CH_3_ of backbone and CH_3_ of side chain). SEC (5 mM NH_4_BF_4_ in DMF) *M*_n_ = 6.4 kg mol^–1^, *Đ* = 1.11.

For irradiation of the pHPMA oligomers, the polymer was dissolved in methanol at 2 mg mL^–1^ and subjected to incubation at 37 °C and light irradiation at 100% power for 18 h prior to MALDI-ToF analysis.

## Results and discussion

### Construction and comparison of isothermal phase diagrams

In order to compare the results from other reports in the literature for similar systems, we first synthesized a PEG macro chain transfer agent (mCTA) with the same molecular weight (5 kg mol^–1^) as the one used in the studies by the groups of Warren and Armes^34^ and Tan, Sumerlin and Zhang^26^ (PEG_113_) using EDC coupling chemistry and an acid functionalized small molecule CTA. A commercially available monomer, HPMA, was used as the core-forming block. Note that this monomer was a mixture of 2-hydroxypropyl methacrylate and 2-isopropyl methacrylate and so the core-forming block is strictly a copolymer of the two isomers (Fig. S1†).^35^ Isothermal phase diagrams were constructed at 37 °C for each of the polymerization techniques (routes A and B). In the photoinitiated system (route A), a 400–410 nm bulb operating at 800 mW was used. Based on the work by Bruns and coworkers,^36^ a commercially available water soluble photoinitiator, 2-hydroxy-4′-2-(hydroxyethoxy)-2-methylpropiophenone (PP-OH), was used as a radical source to initiate the reaction under light irradiation at 405 nm. However, further investigation showed that this particular photoinitiator was not responsible for the initiation of the PISA and instead, the mechanism proceeded *via* an initiator-free photo-RAFT process, as has been described widely for trithiocarbonates.^37^–^39^ Crucially, we observe no difference between formulations irradiated in the presence or absence of PP-OH, and that PP-OH does not show any absorption at the irradiation wavelength of 400–410 nm. For the thermally initiated system (route B), an azo-containing thermal initiator, 2,2′-azobis[2-(imidazolin-2-yl)propane]dihydrochloride, was used. Though this initiator is ionic, the amount of initiator-derived PHPMA homopolymer is expected to be negligible, as the number of initiator-derived chains from a RAFT process is typically extremely low,^40^ and therefore the polymer from the initiator-free photoinitiated formulations and the thermally initiated formulations were designed to contain identical PEG-*b*-HPMA diblock copolymers. The pH of a representative formulation (PEG_113_-*b*-HPMA_300_ at 10 wt% HPMA) was measured to be for 5.2 both routes A and B. It can be seen in Fig. S2† that the kinetics of the photoinitiated sample (route A) was approximately five times faster than the thermally initiated sample (route B) in our study. In each case, there was an inflection point in the kinetic plot at around 17% conversion where the reaction rate vastly increases as a result of the formation of the particles, however this point was reached after around 15 min in route A and 180 min in route B. Each of the polymerizations used to construct the phase diagrams had very high conversion, such that the final monomer concentration was below 1 wt% in the final formulation, as shown in Table S1.† Analysis of a representative sample of polymers formed at 10 wt% HPMA showed that the molecular weight distributions determined by the refractor index (RI) trace were almost identical in each case (Fig. S3-I and S3-II†). Additionally, SEC data throughout the polymerization process were comparable between the two polymerization techniques (Fig. S3-III and S3-IV†).

**Fig. 1:**
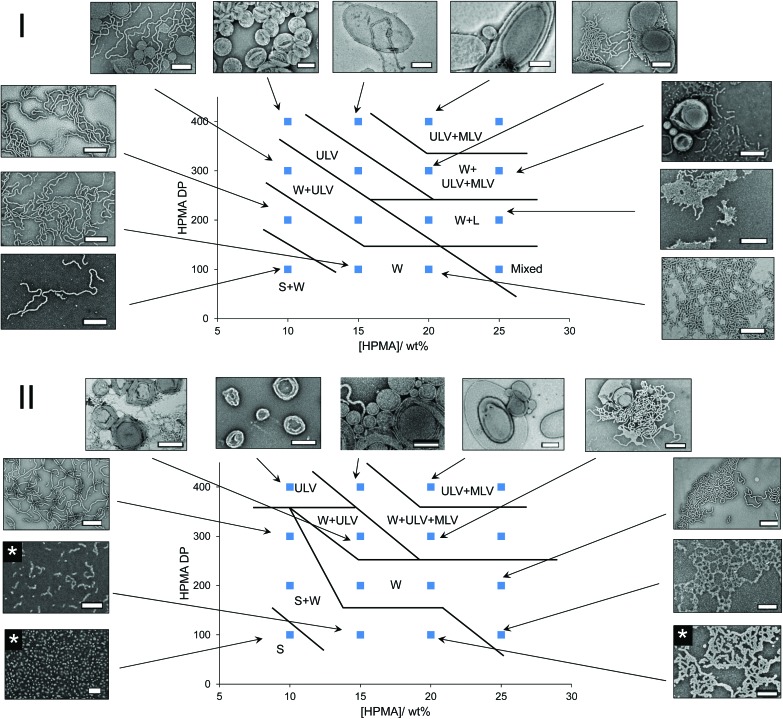
Phase diagrams of PISA formulations synthesized by routes A (I) and B (II) with accompanying stained TEM images. Scale bars represent 0.5 μm, except where marked with an asterisk, in which case the scale bar represents 0.2 μm. Key: S = spheres, W = worms, L = lamellae, ULV = unilamellar vesicles, MLV = multilamellar vesicles.

The two phase diagrams are shown in Fig. 1, with additional TEM and light scattering data included in Fig. S4–36.† As can be seen, our findings suggested that route A, which showed faster reaction kinetics, largely formed higher order morphologies than the thermally initiated system at the same block ratios and concentrations. A few examples are highlighted here. Considering PEG_113_-*b*-PHPMA_100_ at 10 wt% HPMA, contrary to the thermally initiated PISA which yielded predominantly spherical micelles, the photoinitiated PISA formulation gave a mixed phase of spheres with a significant population of long worms. Increasing the DP to 300 at the same concentration, a mixed phase of long worms with spherical unilamellar vesicles were formed by route A, whereas in the route B formulation, a mixed phase of spheres and long worms could be observed. Similarly, pure worm phases were obtained for PEG_113_-*b*-HPMA_100_ formed at 15 and 20 wt% by route A, whereas a mixture of spheres and worms were formed in route B.

For PEG_113_-*b*-HPMA_400_ formed at 10 wt% HPMA, it was observed that unilamellar vesicles were formed, regardless of the preparation technique. In each of these particular formulations, the particles’ size distributions were unimodal as assessed by TEM and DLS analysis for these near-identical formulations. Particle counting analysis was performed on the TEM images, which revealed that vesicles formed by route A had slightly larger diameters and polydispersity than those formed by route B (Fig. S37†).

One outlying data point exists on the photoinitiated phase diagram, which is that of PEG_113_-*b*-HPMA_100_ formed at 25 wt% HPMA. Here, a mixture of spheres, short worms, and both uni- and multilamellar vesicles were obtained. The presence of certain morphologies, particularly spheres, would not be expected to fall in this region of the phase diagram. However, we attribute this discrepancy to the high solution viscosity of the formulation, owing to the high solids content (31 wt% solids), coupled with an increase in reaction kinetics, which prevented homogeneous phase transitions from occurring on the short timescale of the photoinitiated PISA. To some extent this explains why the thermally initiated PISA did not show these unexpected, mixed morphologies at the same concentration because the monomer conversion, and therefore morphological evolution, occurred more slowly, which allowed for homogeneous phase transitions. The total solids content decreases with increasing HPMA DP as the contribution from the PEG mCTA decreases in these formulations.

It is important to stress that pure phases could be obtained by both methods and that by carefully tuning the conditions, for instance the block ratio and concentration, we believe it is possible to obtain very similar morphologies regardless of the technique. However, the morphologies formed will occupy very different regions of the phase diagram depending on the preparation pathway. These initial results are somewhat analogous to the findings in Tan, Sumerlin, Zhang and co-workers’ study on the effect of temperature using constant reaction kinetics.^26^ Here they found that higher order structures were formed at higher temperatures for identical formulations prepared using photoinitiated PISA, the reaction kinetics of which were not affected by reaction temperature.

### The influence of light intensity on the final PISA morphology

**Fig. 2:**
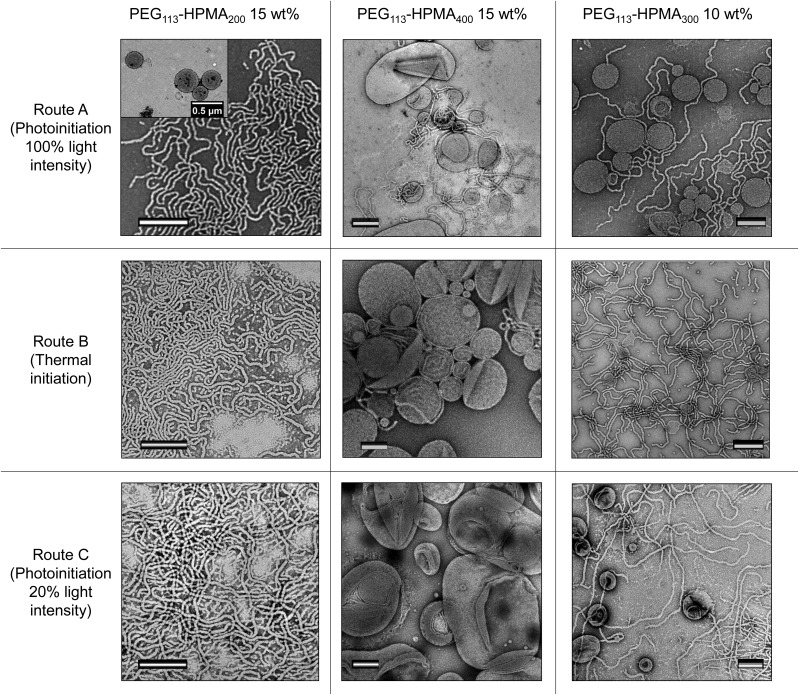
Stained TEM images of particles from various formulations formed from route C (bottom row), shown in comparison to identical formulations formed from routes A (top row) and B (middle row). The scale bars all represent 500 nm.

In order to further understand the influence of the kinetics of particle formation in the PISA process, a number of model formulations were synthesized using a lower light intensity (route C). Because the rate of polymerization for the 100% light intensity photoinitiated PISA (route A, 800 mW power) was approximately five times faster than the thermally initiated PISA (route B), roughly 20% of the light intensity was used in route C (roughly 160 mW power), by use of a dimmer switch, in order to attempt to match the polymerization rate of the thermally initiated PISA (route B). A kinetic study showed that our approach worked well, with the kinetic data for routes B and C overlaying (Fig. S38-I†), as a rough approximation. Additionally, we observed similar *M*_n, SEC_ and *Đ* values at various conversions throughout the polymerization to that of routes A and B (Fig. S38-II†). Certain formulations were chosen for route C, two of which showed a significant difference in morphology between the routes A and B, namely PEG_113_-*b*-HPMA_300_ formed at 10 wt% and PEG_113_-*b*-HPMA_200_ formed at 15 wt%, and one of which showed similar morphologies between routes A and B, namely PEG_113_-*b*-HPMA_400_ formed at 15 wt% (Fig. 2). The structures were investigated after 5.5 h (route C) and were further irradiated with light at 37 °C overnight (route C + F). Comparing the structures left overnight to those formed after 5.5 h revealed no significant change in morphology by irradiating for longer periods at this light intensity (Fig. S39†).

It was revealed from this study that the PEG_113_-*b*-HPMA_200_ formulation at 15 wt% resulted in a pure phase of worms when formed by route C (Fig. 2, bottom LHS). This was in closer agreement with the structures formed by route B (worms) than route A (worms and unilamellar vesicles), both of which are shown in Fig. 2 for comparison. This initial result was promising as it showed that the structures formed were dictated by their rate of formation, since routes B and C have near-identical rates of polymerization, and therefore rates of particle formation. PEG_113_-*b*-HPMA_400_ at 15 wt% formed large uni- and multilamellar vesicles by route C (Fig. 2, bottom center). This can be understood by the fact that the particle formation occurs much more slowly and so chain exchange and fusion events can occur to a greater extent thereby facilitating the formation of larger structures. However, a minor population of very small unilamellar vesicles was also observed indicating that some kinetic trapping was still occurring during the PISA process. In this example, unlike in the formulation formed by route B, no worm morphologies were observed and the vesicle structures formed were significantly larger in route C. The final test formulation was further evidence that reaction kinetics was not the sole parameter at play in determining the final morphology formed. PEG_113_-*b*-HPMA_300_ at 10 wt% formed by route C somewhat unexpectedly formed a mixture of unilamellar vesicles and long worms, similar to those formed by route A and in contrast to those formed by route B. This indicated that the structures formed in this example were not simply a result of the reaction kinetics as near-identical reaction kinetics (routes B and C) using two different preparation pathways (*i.e.* thermal and light initiation) resulted in different final morphologies. We hypothesized that this intriguing result could unveil the differences between the phase diagrams and help further understand the influence of factors on the nanostructure morphology other than the reaction kinetics.

### The influence of post-synthetic light and heat irradiation on the pre-formed PISA morphologies

Following the somewhat unexpected results discussed in the previous section, the influence of post-synthetic light and heat irradiation on the pre-formed PISA particles was investigated. For these experiments, PEG_113_-*b*-HPMA_300_ was used as a model block ratio, as this block ratio showed the most obvious dependence on numerous factors, as seen in comparing routes A, B and C (Fig. 2, RHS column). Additionally the exact kinetics of this specific formulation had been determined in each case. The formulation at 10 wt% from both routes A and B were left overnight at 37 °C either with (route D) or without (route E) irradiation at 100% light intensity. It should be noted that a control experiment with deionized water fitted with an internal temperature sensor did not show any appreciable increase in temperature from the light irradiation, confirming that the experiments were carried out at identical temperatures. Note also that the PISA formulations had already reached full conversion and that no degassing of the solutions took place before treatment.

**Fig. 3:**
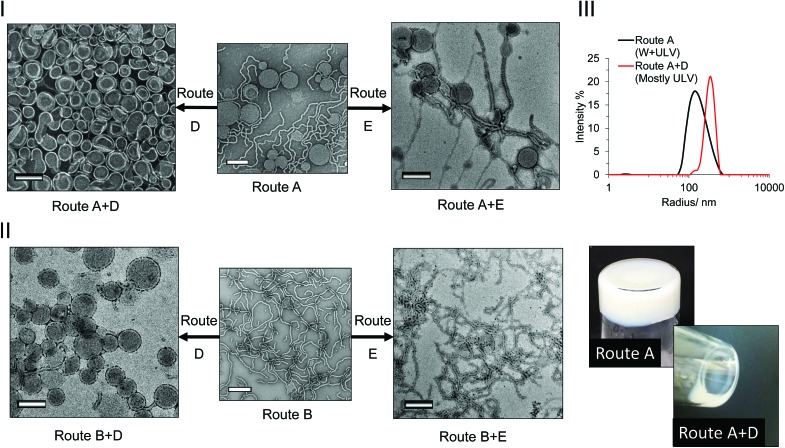
Post-synthetic heat and light irradiation experiments. Stained TEM images of PEG_113_-*b*-HPMA_300_ formulations obtained at [HPMA] = 10 wt% by route A (I) or route B (II), treated by route D (LHS) or route E (RHS). The scale bars represent 500 nm. III: DLS analysis (top) and photographs (bottom) of formulations formed by route A and routes A + D. Key: route A – photoinitiated PISA at 100% light intensity at 37 °C. Route B – thermal initiation at 37 °C. Route D – photoirradiation at 100% light intensity at 37 °C for 18 h. Route E – incubation at 37 °C for 18 h.

The results from these experiments revealed that no appreciable change in morphology resulted from heat irradiation alone (route E). The photoinitiated formulation treated with just heat (route A + E) remained as a mixed phase of unilamellar vesicles and worms (Fig. 3-I, RHS and Fig. S40†), whereas the formulation formed by route B + E was found to predominantly contain worms (Fig. 3-II, RHS and Fig. S41†), in fair agreement with the morphologies found prior to the additional heating (Fig. 3-I and 3-II center images). In the experiments with both light and heat irradiation (route D), there was a noticeable difference in the physical form of the formulation. The formulations were found to undergo a transition from a solid gel to a flowing white liquid (Fig. 3-III, bottom). TEM analysis of the formulation formed by route A + D revealed that an almost pure phase of unilamellar vesicles had been formed in the formulation after light irradiation overnight, with only a very minor population of worms remaining (Fig. 3-I, LHS and Fig. S40†). DLS analysis also revealed a shift in the radial distribution towards larger structures, in agreement with the occurrence of a morphological transition (Fig. 3-III, top). Perhaps even more surprisingly, the formulation formed by route B + D, which did not contain any vesicle structures prior to light irradiation, was found to exist as an almost pure phase of unilamellar vesicles after overnight light irradiation (Fig. 3-I, LHS and Fig. S41†).

**Fig. 4:**
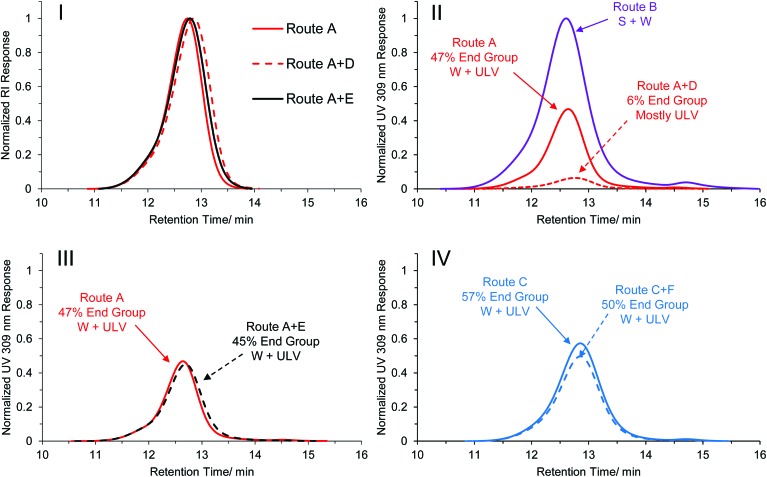
SEC traces of PEG_113_-*b*-HPMA_300_ at [HPMA] = 10 wt% formed by various synthetic and post-synthetic routes. I: Normalized RI traces of formulations formed by routes A, A + D and A + E. II: Normalized 309 nm traces of formulations formed by routes A, B and A + D. III: Normalized 309 nm traces of formulations formed by routes A and A + E. IV: Normalized 309 nm traces of formulations formed by routes C and C + F. Routes C and C + F are analogous experiments to A and A + D but at the lower light intensity irradiation. In each of the UV traces shown in II, III and IV the traces are normalized relative to the formulation formed by route B. The end group fidelity relative to route B is shown, along with the observed morphology determined by TEM.

**Fig. 5:**
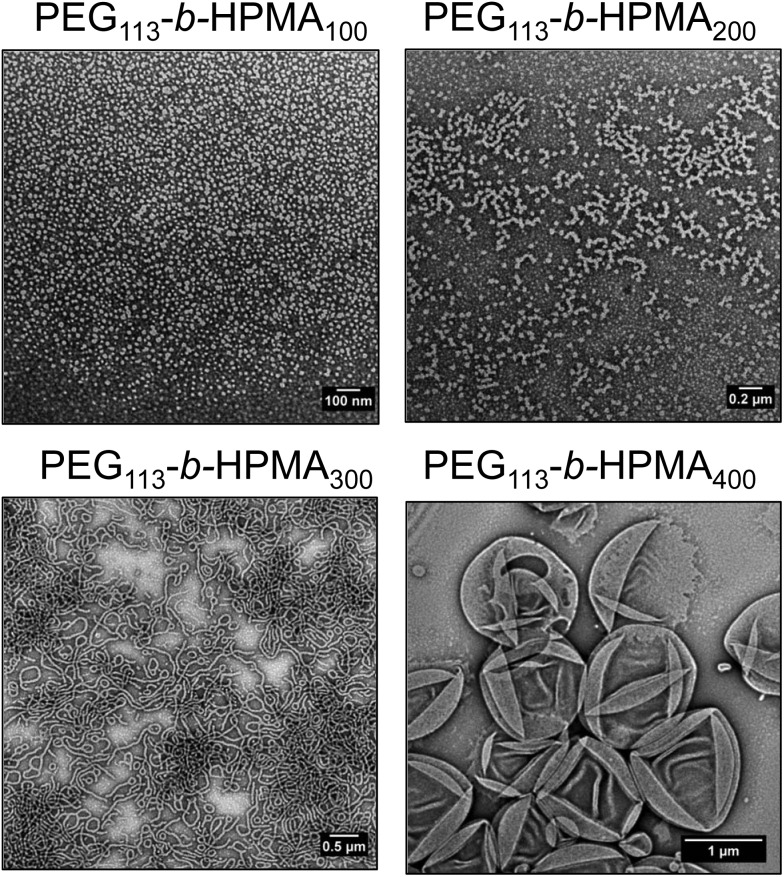
Stained TEM images of equilibrium structures of diblock copolymers formed at 37 °C and 1 mg mL^–1^. The block ratio for each sample is indicated above the corresponding TEM image.

This result indicated that the light itself had an influence on the final morphology obtained, and therefore may explain to some extent the differences observed in the original PISA phase diagrams. The PEG_113_-*b*-HPMA_300_ polymers formed at 10 wt% prepared by all synthetic and post-synthetic routes were comparatively analyzed by SEC in order to elucidate any differences caused by the light irradiation. No appreciable differences were observed between the samples when comparing the polymers’ RI traces, indicating that no appreciable cross-linking or degradation of the side chains or backbone had occurred as a result of the photoirradiation (Fig. 4-I). However, the normalized UV traces measured at 309 nm revealed that those formed by light-mediated PISA (routes A and C, Fig. 4-II, 4-III and 4-IV, solid red and blue traces respectively) retained only roughly half of their end group relative to those formed by thermal initiation (route B, Fig. 4-II, purple trace). Additionally, the formulation formed by route A + D (ULVs) were greatly reduced (Fig. 4-II, dashed red trace) compared to the original untreated sample formed by route A (ULV + W, Fig. 4-II, solid red trace). This indicated that almost 90% of the trithiocarbonate end group, relative to that formed by route A had cleaved off the end of the polymer chains in the presence of light and the absence of monomer (route A + D). In contrast, the formulation formed by route A + E contained 96% of its end group after heat treatment alone (Fig. 5-III) and as such no morphology change was observed. In those formed by route C (Fig. 4-IV, solid blue trace), the polymers retained a slightly higher end group fidelity than those formed by route A (Fig. 4-I, solid red trace). Additionally, after overnight irradiation at the lower light intensity, the polymers retained 88% of their end group relative to those formed by route C alone, which is in agreement with the observation that irradiating with a lower light intensity overnight did not result in a morphological transition (Fig. S39†).

Considering the evolution of morphologies across Fig. 4-II, III and IV (denoted next to the arrows for each trace), there appears to be a distinct trend in the morphologies formed with the degree of end group fidelity, with those polymers with lower end group fidelity having a tendency to form higher order structures across this series of synthetic and post-synthetic routes. This is further evidenced by the fact that when no morphology transitions occur, there is a high level of end group retention (Fig. 4III and IV). We therefore propose that differences in both reaction kinetics and end group fidelity lead to differences in the phase diagrams in the original thermally initiated and photoinitiated systems. Light-mediated end group removal of trithiocarbonates under non-inert atmosphere are recently being realized in the literature^41^–^43^ and since photolysis of trithiocarbonates is a key step in light-mediated RAFT processes,^37^,^38^,^44^,^45^ it is not unreasonable that this mechanism could be important here. Note that this is in contrast to the findings by Warren, Armes and co-workers,^46^ the only report of end group removal after a PISA process to our knowledge, who found that end group removal using hydrogen peroxide did not induce a change in morphology. However, since the two end group removal processes are so distinct, the final end groups after trithiocarbonate removal may be very different between our report and that by Warren, Armes and co-workers, leading to overall different behavior. We also observed similar end group retention when the solution was degassed, compared with when the solution was irradiated in a non-inert atmosphere (Fig. S42†). Although it is unlikely that the loss of the ethyl trithiocarbonate end group alone is enough to drive a morphological transition in polymers of this high molecular weight, it is possible that any change in end group (*e.g.* to an ionic or hydrophilic species) could allow for an increase in chain exchange dynamics and overcome the barrier of chain rearrangement, thereby facilitating a morphology transition. We attempted to investigate the molecular structural changes associated with the end group under light irradiation by synthesizing a model PHPMA oligomer (DP = 20). This was fractionated by preparative SEC and short oligomers were studied by MALDI-ToF MS. The initial major distribution was in close agreement with a polymer containing both R and Z groups of the CEPA CTA (Fig. S43,† black trace). After light irradiation in methanol, the new distribution revealed a mass difference of 154 Da (Fig. S43,† blue trace), however we were unable to quantitatively determine the structure of the new end group from this analysis. ^1^H NMR spectroscopy of the irradiated oligomers showed a disappearance of the ethylene protons adjacent to the trithiocarbonate group at 3.31 ppm in acetone-d6 after the light irradiation (Fig. S44†). The post synthetic procedure of route D was repeated with a PEG_113_-*b*-HPMA_300_ formulation that contained other morphologies to see the extent of the transitions that could occur, namely the formulation obtained by route A at 20 wt%, which formed a mixture of worms, unilamellar vesicles and multilamellar vesicles. After light irradiation at 37 °C overnight, a marked reduction in worm structures were observed by TEM, however a transition from multilamellar to unilamellar vesicles was not observed (Fig. S45†). This was somewhat expected by virtue of the fact that both are bilayer structures and so the packing and curvature difference between a multilamellar vesicle and a unilamellar vesicle is not significant enough to induce polymer rearrangement under light irradiation.

### Investigation into the equilibrium morphologies formed at 37 °C

In order to further investigate the formation of higher order morphologies in the formulations formed by route A, as well as the light-mediated morphology change discussed in the previous section, the equilibrium morphologies of PEG_113_-*b*-HPMA_100_, PEG_113_-*b*-HPMA_200_, PEG_113_-*b*-HPMA_300_ and PEG_113_-*b*-HPMA_400_ were assessed. These four diblock copolymers were dissolved in ethanol, a common solvent for both blocks to disassemble the nanostructures, followed by slow addition of the selective aqueous solvent and evaporation of the organic solvent. However this was performed in dilute solution (1 mg mL^–1^) to avoid precipitation of the block copolymers, therefore this experiment was designed to give comparative information between the samples. Fig. 5 shows the evolution of morphologies at equilibrium across the four diblock copolymer ratios investigated, with higher order morphologies being formed by those with larger hydrophobic weight fractions, as expected. PEG_113_-*b*-HPMA_100_ formed a pure phase of spherical micelles. PEG_113_-*b*-HPMA_200_ formed a mixture of short worms and spherical micelles, indicating this polymer was likely close to a thermodynamic phase boundary and a mixture of structures had formed as a result of the polymer's dispersity. PEG_113_-*b*-HPMA_300_ formed worm-like micelles and PEG_113_-*b*-HPMA_400_ formed large unilamellar vesicles, which collapsed to reveal hollow structures when dried onto the TEM grid (Fig. 5).

To demonstrate that removal of the end group during light irradiation drives the formation of new structures, the PEG_113_-*b*-HPMA_300_ at [HPMA] = 10 wt% formulation formed by route A + D after undergoing the morphological transition was dissolved in ethanol before adding water slowly at 37 °C, to obtain the new equilibrium structure for this block ratio after light treatment. It was observed that a significant reduction in worms, the original equilibrium structure, towards spheres and small vesicles had occurred in the newly obtained equilibrium morphology after irradiation (Fig. S46†).

The results from this experiment, though not entirely conclusive, suggests that there is a driving force for the nanostructures to rearrange into a new morphology during light-mediated end group removal and that a lack of control over the end group fidelity during the polymerization could drive the formation of different morphologies resulting in some of the differences observed between the phase diagrams in Fig. 1. The presence of spheres could be a result of a kinetic barrier still not overcome by the preparation method of the equilibrium structures since this morphology was not observed in the formulation formed by route A + D. The degree of polymerization control over the PISA process has been shown by Boyer and co-workers to have a marked effect on the particle morphology,^27^ which is analogous to our findings, whereby control over the end group fidelity can have an influence over the obtained particle morphologies.

## Conclusions

In this study, an insight into the effect of reaction kinetics and the influence of light on the final particle morphologies obtained in a PISA process was gained by construction of isothermal phase diagrams by two preparation pathways, photoinitiated PISA and thermally initiated PISA. Investigation revealed that a loss of end group, observed in the photoinitiated PISA, leads to the formation of higher order structures, to which we attribute some of the differences observed between the photoinitiated and thermally initiated PISA phase diagrams. It was shown that the loss of end group upon further post-synthetic light irradiation can additionally cause a shift in morphology towards higher order structures upon extended periods of high intensity light irradiation. Whilst pure morphological phases could be obtained in both cases, our study highlights that thermally initiated and photoinitiated PISA can lead to very different obtained morphologies for any given formulation and that pure phases may exist in different regions of the phase diagram depending on the polymerization method. Additionally, where the morphologies are in agreement for identical block ratios with near-identical molecular weight distributions, the polymers may not be chemically identical by virtue of the differences in end-group fidelity, which may be important for applications where the ω-end group is required. We anticipate that the utilization of photoinitiated PISA will become more prominent in the future, owing to its ability to form predictable morphologies within minutes under very mild reaction conditions and we hope that these findings contribute to a deeper understanding of this industrially relevant process.

## Supplementary Material

Supplementary information
