# Supplementary material for: Comparison of photo- and thermally initiated polymerization-induced self-assembly: a lack of end group fidelity drives the formation of higher order morphologies
Source: Polym Chem. 2017 Apr 19;8(18):2860–71. doi: 10.1039/c7py00407a (PMC5718300; doi:10.1039/c7py00407a)
Supplement: Supplementary file 1 [file PY-008-C7PY00407A-s001.pdf]

Electronic Supplementary Material (ESI) for Polymer Chemistry.
This journal is © The Royal Society of Chemistry 2017

Supplementary Information

Comparison of photo- and thermally initiated polymerization-induced self-assembly; lack of end group fidelity drives the formation of higher order morphologies

Lewis D. Blackman,[a] Kay E. B. Doncom,[a] Matthew I. Gibson[a,b] and Rachel K. O'Reilly[a]**

[a] *Dept. of Chemistry, University of Warwick, Gibbet Hill Road, Coventry, CV4 7AL, UK*

[b] *Warwick Medical School, University of Warwick, Gibbet Hill Road, Coventry, CV4 7AL, UK*

Contents

Supplementary ^{1}H NMR spectroscopy data 2

Kinetics and conversion data for formulations formed by routes A and B 2

Supplementary SEC data for routes A and B 4

Supplementary TEM data for formulations formed by route A 5

Supplementary TEM data for formulations formed by route B 13

Supplementary DLS data for formulations formed by routes A and B 21

Histograms of PEG_{113}-*b*-$HPMA_{400}$ unilamellar vesicles 22

Supplementary data for formulations formed by route C 23

Supplementary data for the post-synthetic irradiation experiments 24

Equilibrium structure of PEG_{113}-*b*-$HPMA_{300}$ formed by route A+D. 29

Supplementary ^{1}H NMR spectroscopy data

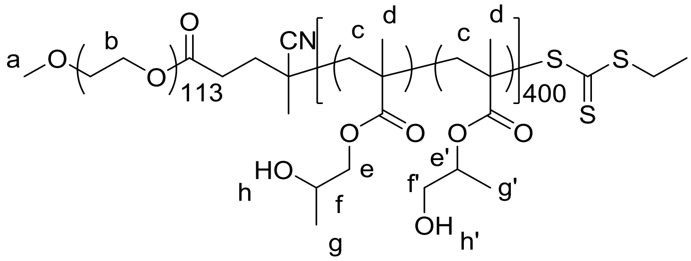

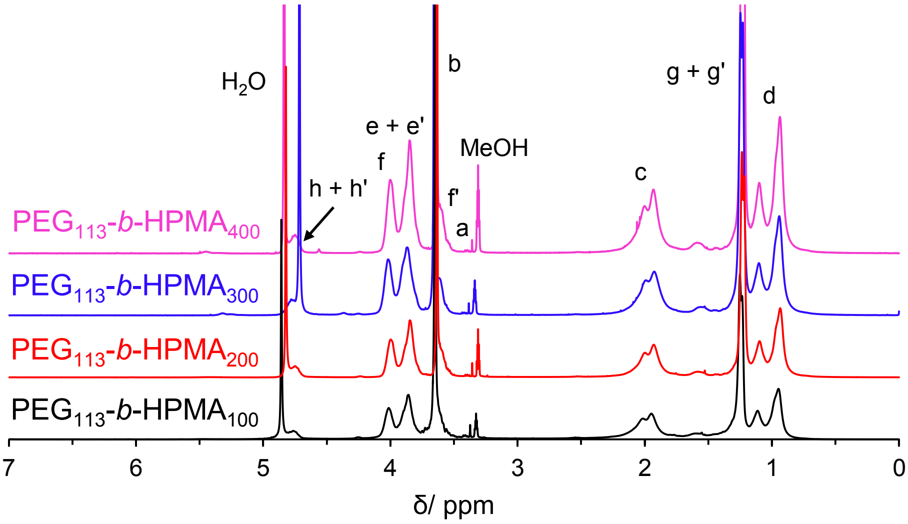

Fig. S1. Representative ^{1}H NMR spectra of PEG_{113}-$PHPMA_x$ diblock copolymers formed at 10 wt% HPMA by route B analyzed at 300 MHz in CD_3OD. The spectral intensities were normalized to the PEG mCTA at 3.63 ppm (signal "b").

Kinetics and conversion data for formulations formed by routes A and B

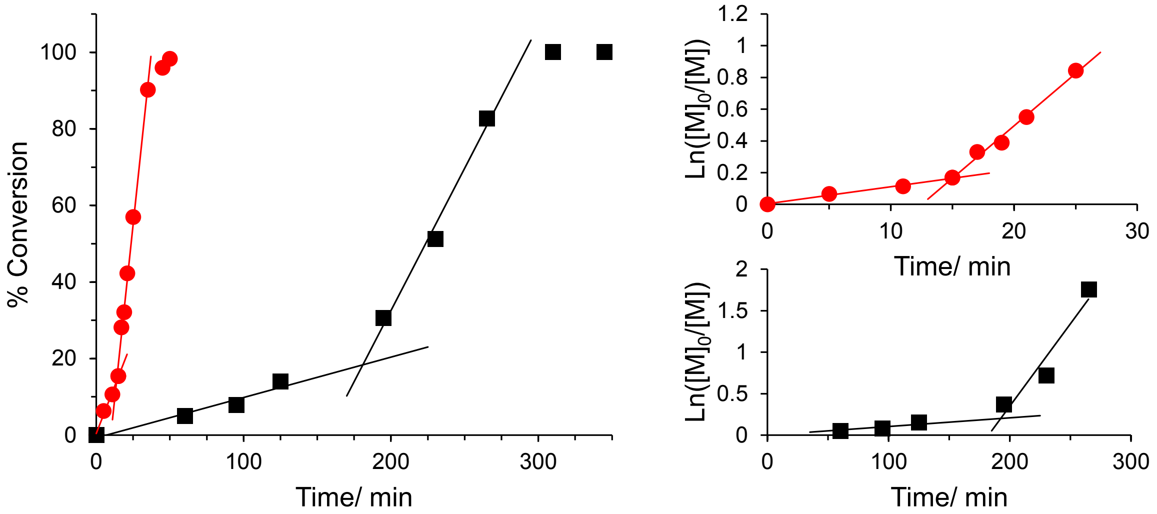

Fig. S2. Kinetic plots of formulations formed by routes A (red circles) and B (black squares). In each case an inflection point has been indicated by the intercept of the two gradients, which shows the onset of particle aggregation.

Method	PHPMA Target DP	[HPMA]/ wt%	Conversion	Method	PHPMA Target DP	[HPMA]/ wt%	Conversion
Route A	100	10	>99%	Route B	100	10	>99%
Route A	100	15	>99%	Route B	100	15	>99%
Route A	100	20	>99%	Route B	100	20	>99%
Route A	100	25	>99%	Route B	100	25	>99%
Route A	200	10	>99%	Route B	200	10	>99%
Route A	200	15	>99%	Route B	200	15	>99%
Route A	200	20	>99%	Route B	200	20	>99%
Route A	200	25	>99%	Route B	200	25	>99%
Route A	300	10	>99%	Route B	300	10	>99%
Route A	300	15	>99%	Route B	300	15	>99%
Route A	300	20	97%	Route B	300	20	>99%
Route A	300	25	98%	Route B	300	25	>99%
Route A	400	10	>99%	Route B	400	10	>99%
Route A	400	15	>99%	Route B	400	15	>99%
Route A	400	20	97%	Route B	400	20	>99%
Route A	400	25	99%	Route B	400	25	>99%

Table S1. Summary of final conversions as assessed by ^{1}H NMR spectroscopy for the 100% light intensity photoinitiated (route A) and thermally initiated (route B) PISA formulations.

Supplementary SEC data for routes A and B

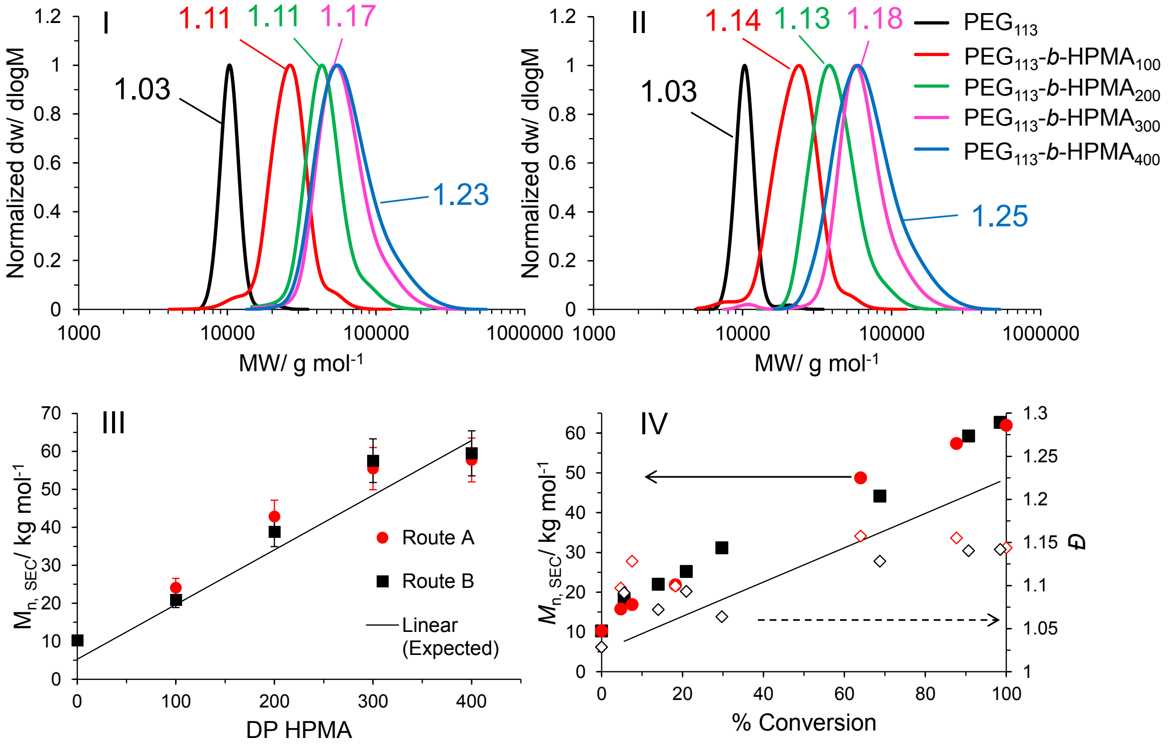

Fig. S3. Representative RI SEC traces for PEG_{113}-*b*-$HPMA_x$ diblock copolymers formed at 10 wt% HPMA using 5 mM NH_4BF_4 in DMF as the eluent**.** Data for route A (I) and route B (II) are shown along with the *Đ* values. Key: In each case, PEG_{113} (black trace), PEG_{113}-*b*-$HPMA_{100}$ (red trace), PEG_{113}-*b*-$HPMA_{200}$ (green trace), PEG_{113}-*b*-$HPMA_{300}$ (magenta trace) and PEG_{113}-*b*-$HPMA_{400}$ (blue trace) are shown. Panel III shows the calculated M_n values for route A (red circles) and route B (black squares) derived diblock copolymers shown in panels I and II, using PMMA standards. Error bars represent 10% error. The black linear trend shows the expected M_n values. Panel IV shows SEC data from the kinetic study of PEG_{113}-*b*-$HPMA_{300}$ formed by routes A (red) and B (black). Left axis: M_n values of polymers formed by route A (red circles) and route B (black squares), the black linear trend shows the expected M_n values. Right axis: *Đ* values of polymers formed by route A (red diamonds) and route B (black diamonds).

Supplementary TEM data for formulations formed by route A

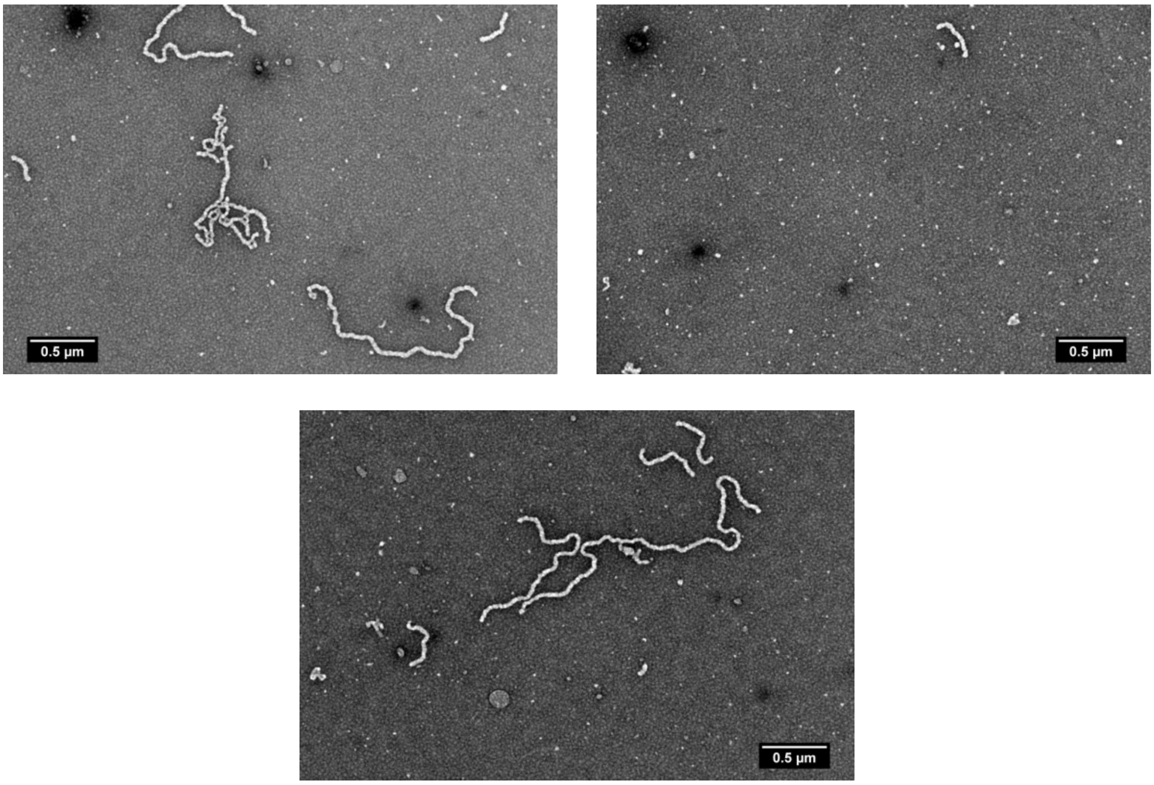

Figure S4. Representative TEM images of PEG$_{113}$-*b*-HPMA$_{100}$ formed at 10 wt% HPMA (S+W).

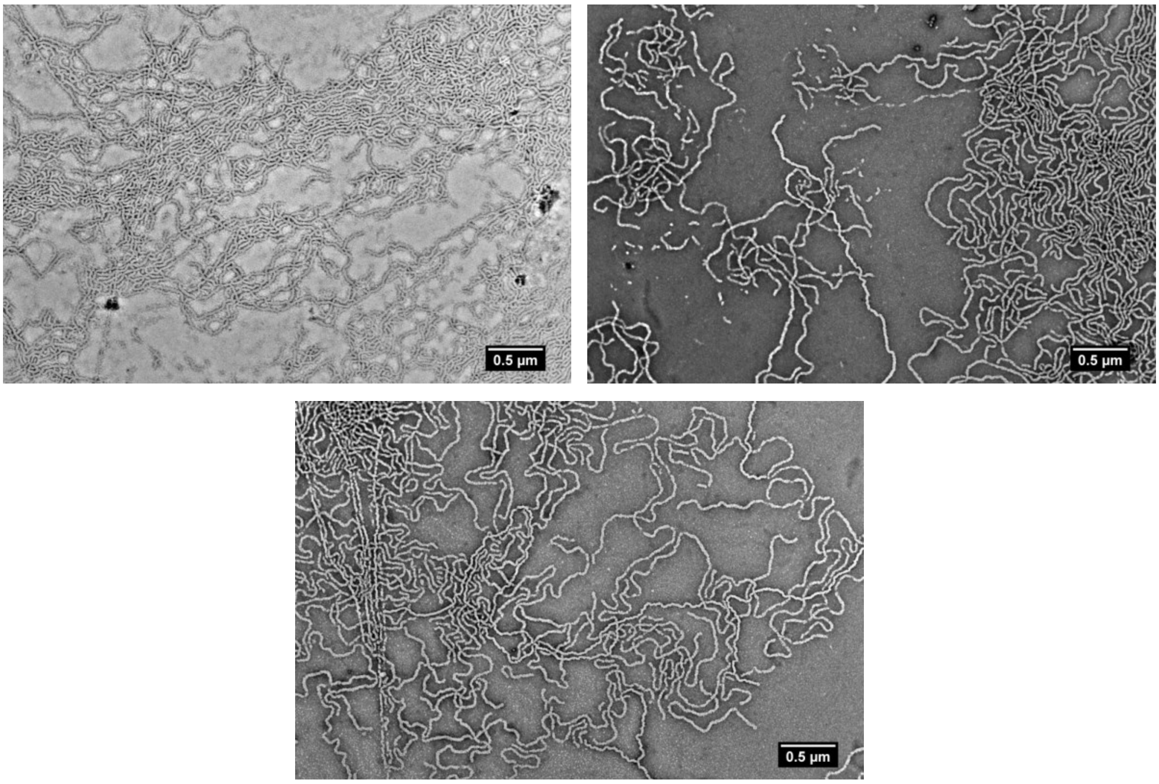

Figure S5. Representative TEM images of PEG$_{113}$-*b*-HPMA$_{100}$ formed at 15 wt% HPMA (W).

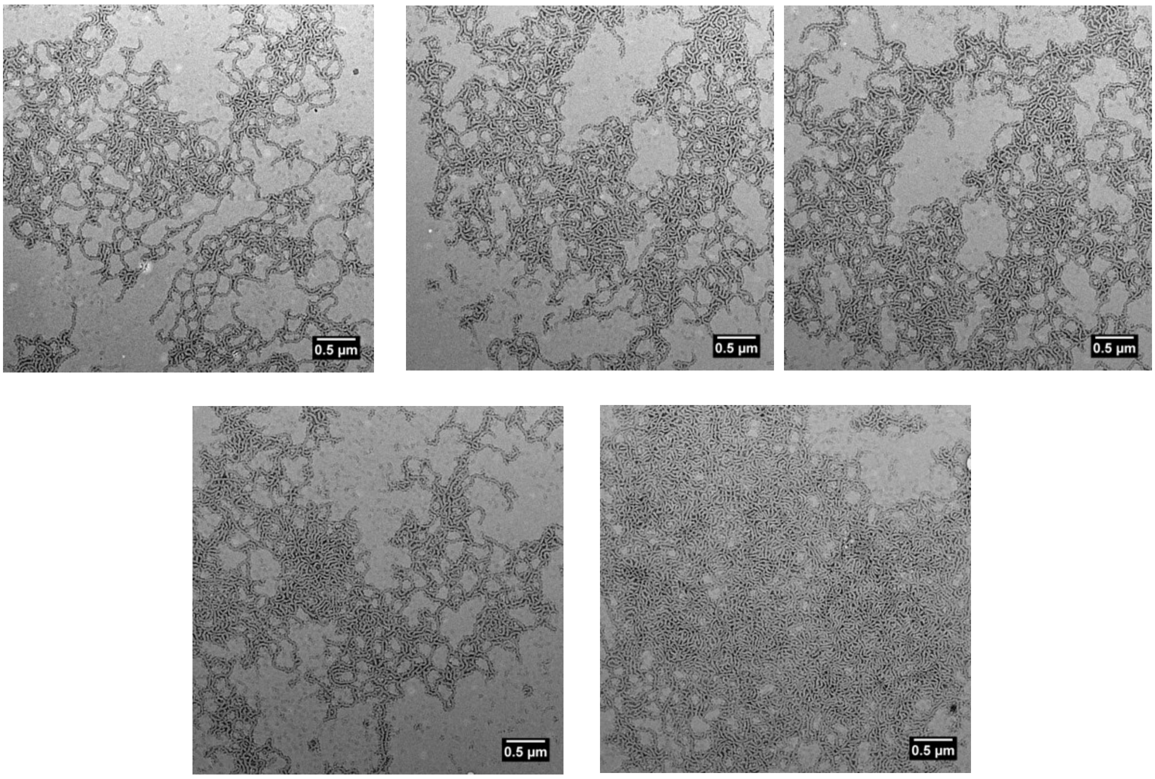

Figure S6. Representative TEM images of PEG_{113}-*b*-$HPMA_{100}$ formed at 20 wt% HPMA (W).

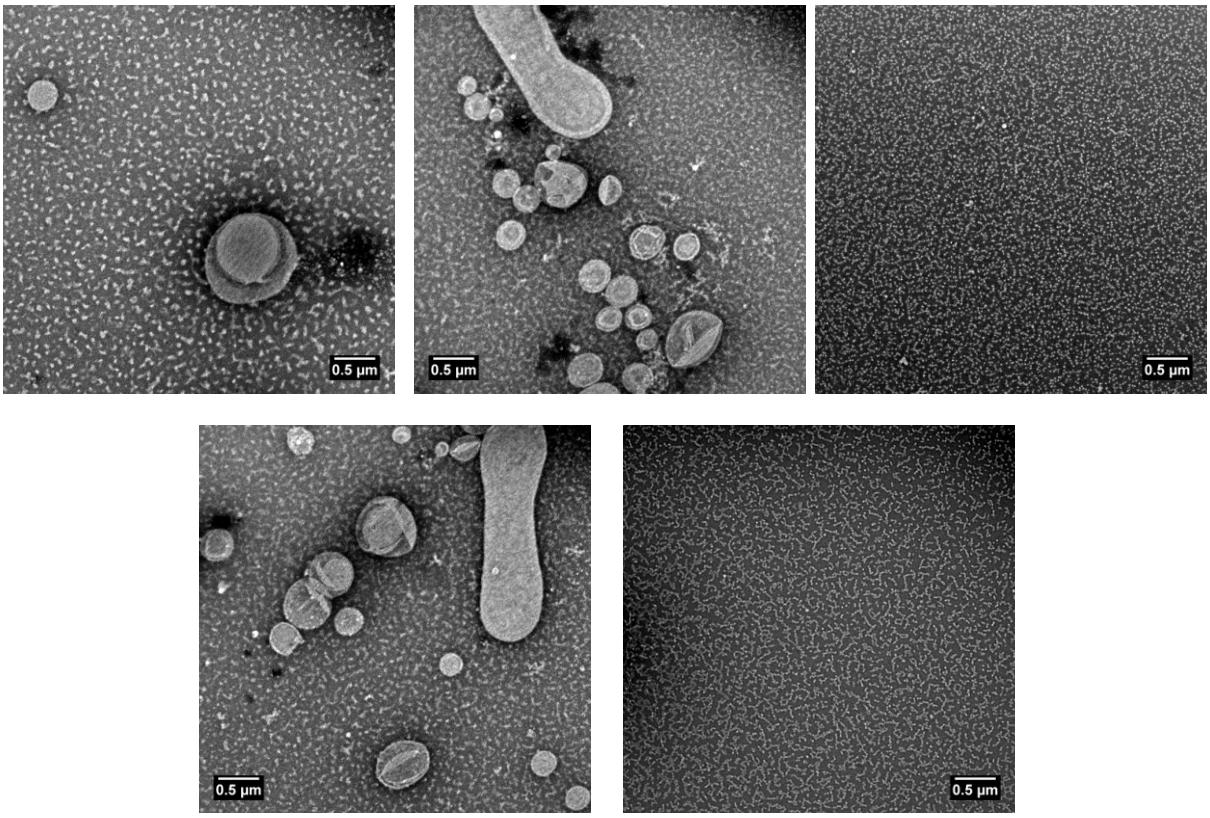

Figure S7. Representative TEM images of PEG_{113}-*b*-$HPMA_{100}$ formed at 25 wt% HPMA (Mixed).

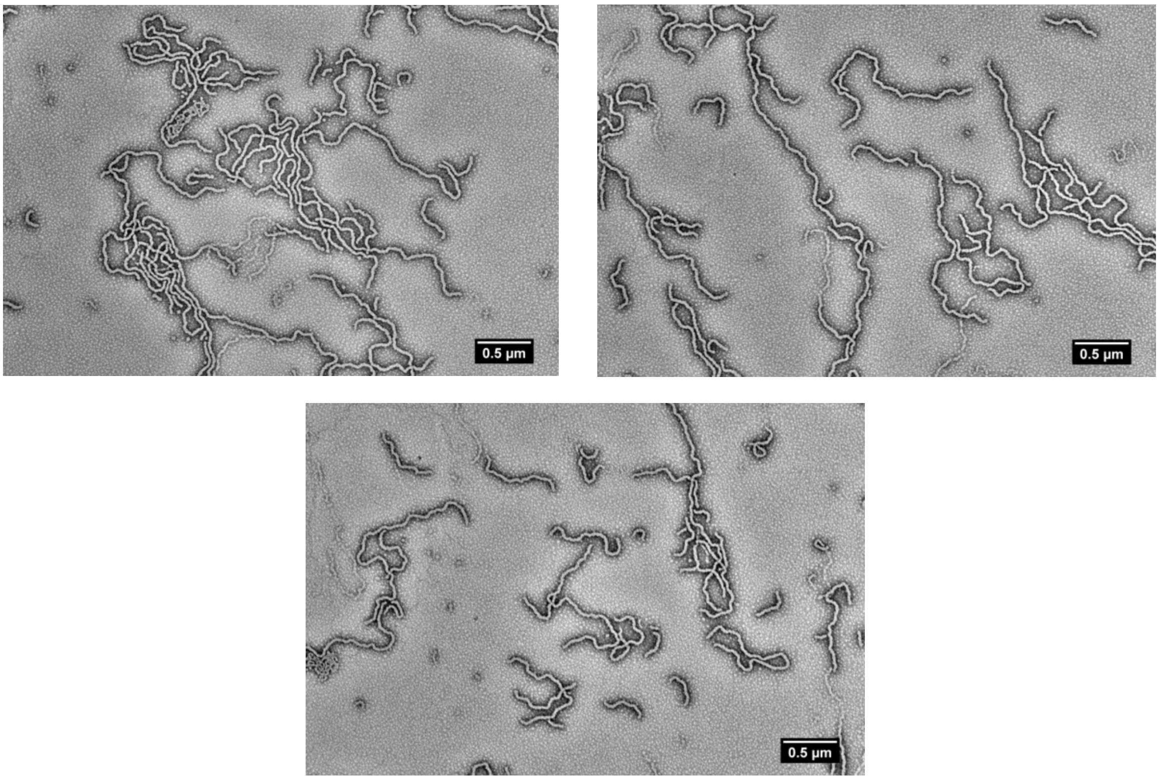

Figure S8. Representative TEM images of PEG_{113}-*b*-$HPMA_{200}$ formed at 10 wt% HPMA (W).

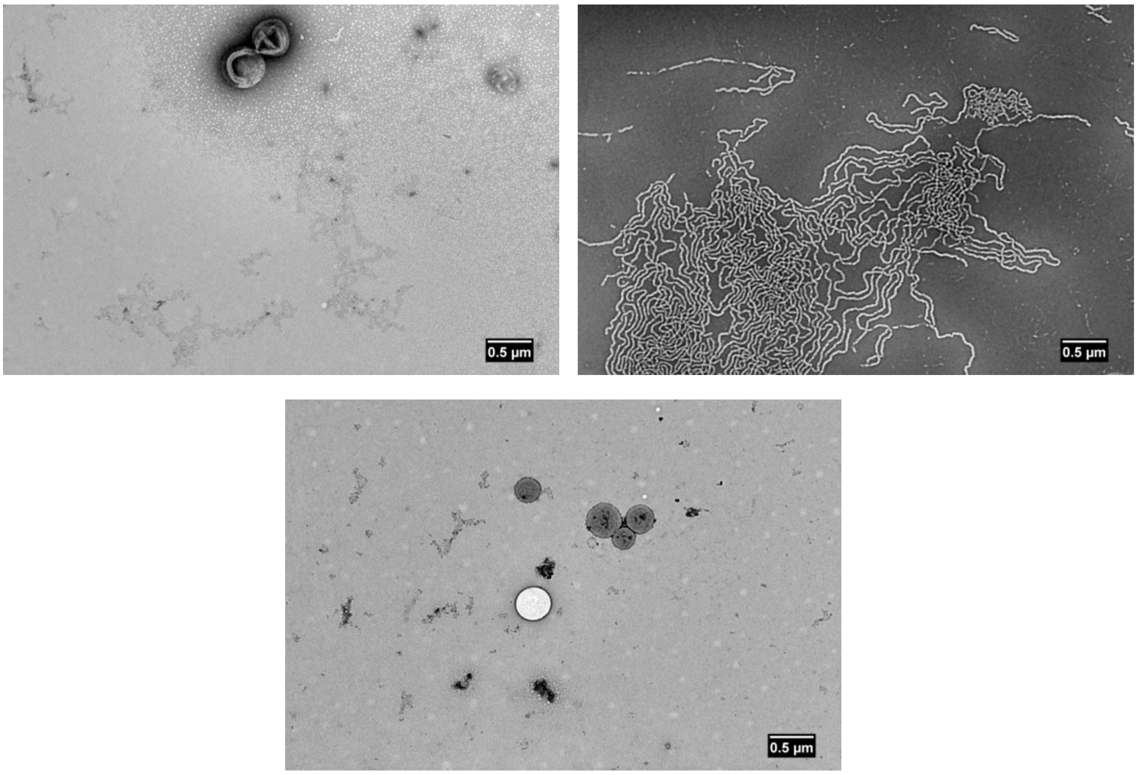

Figure S9. Representative TEM images of PEG_{113}-*b*-$HPMA_{200}$ formed at 15 wt% HPMA (W+ULV).

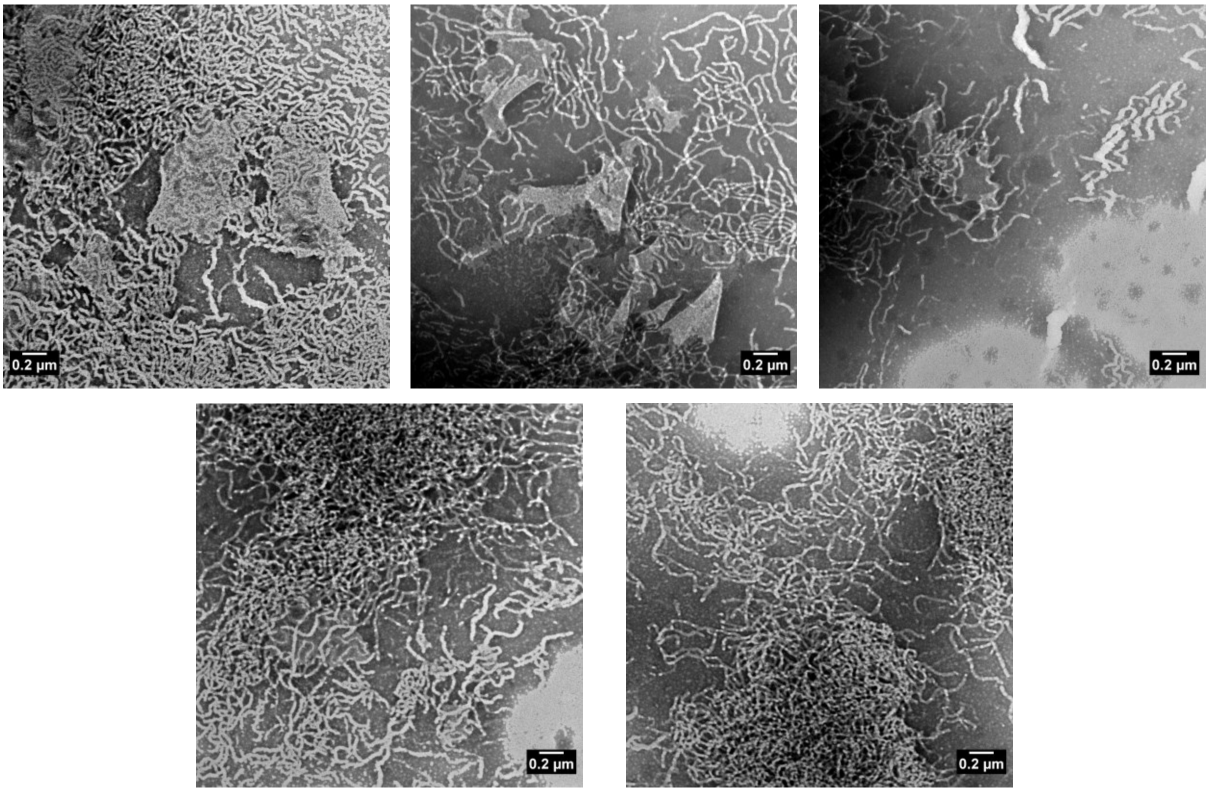

Figure S10. Representative TEM images of PEG_{113}-*b*-$HPMA_{200}$ formed at 20 wt% HPMA (W+L).

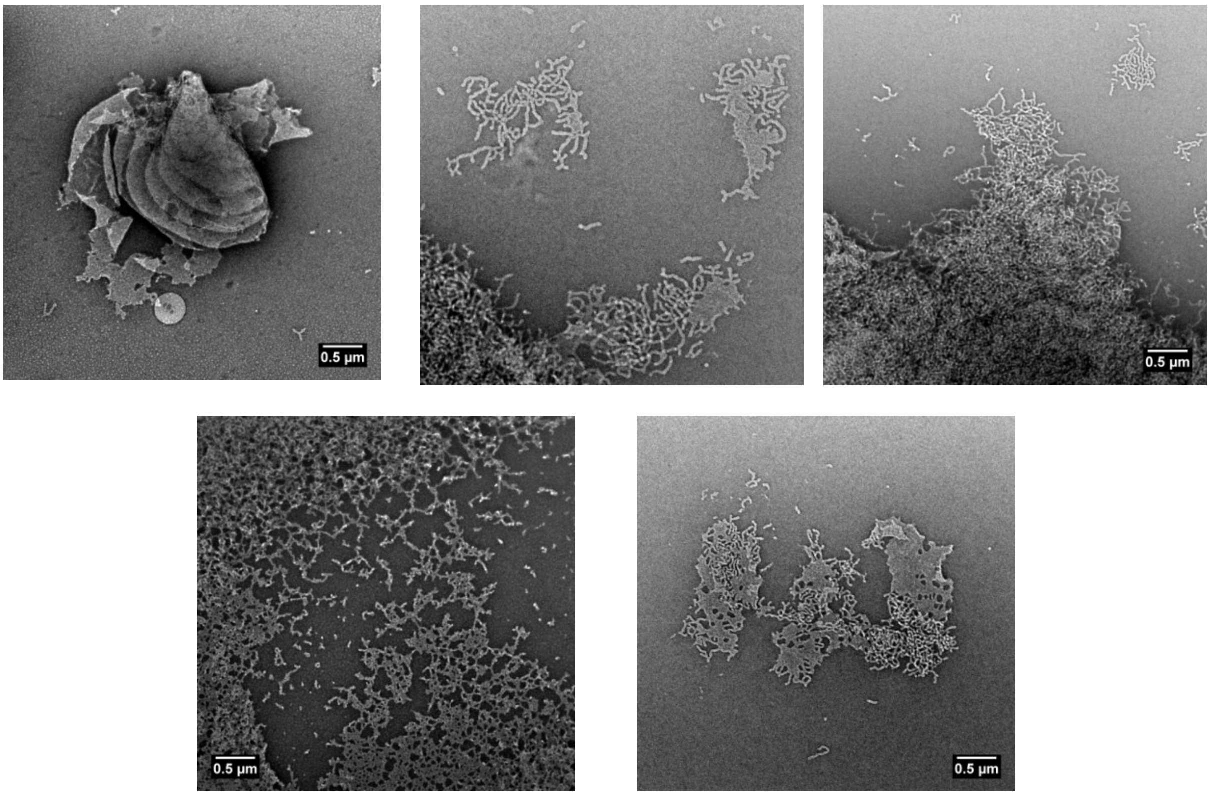

Figure S11. Representative TEM images of PEG_{113}-*b*-$HPMA_{200}$ formed at 25 wt% HPMA (W+L).

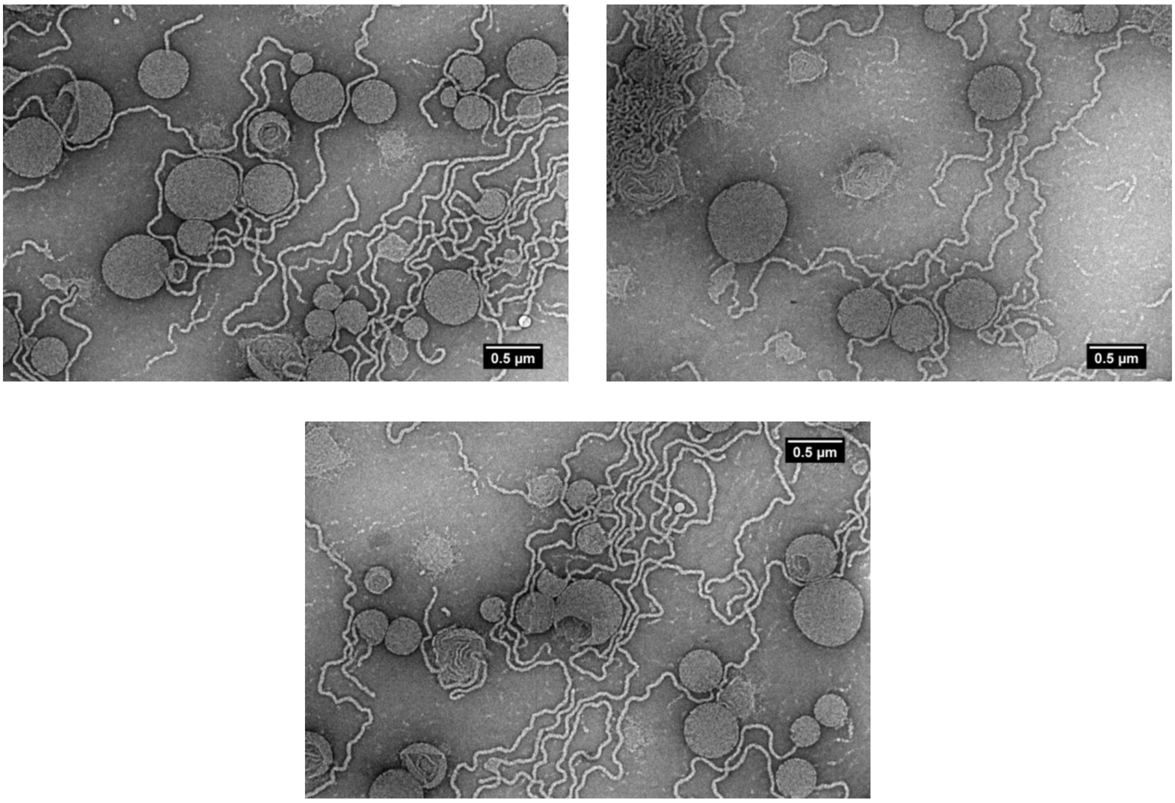

Figure S12. Representative TEM images of PEG_{113}-*b*-$HPMA_{300}$ formed at 10 wt% HPMA (W+ULV).

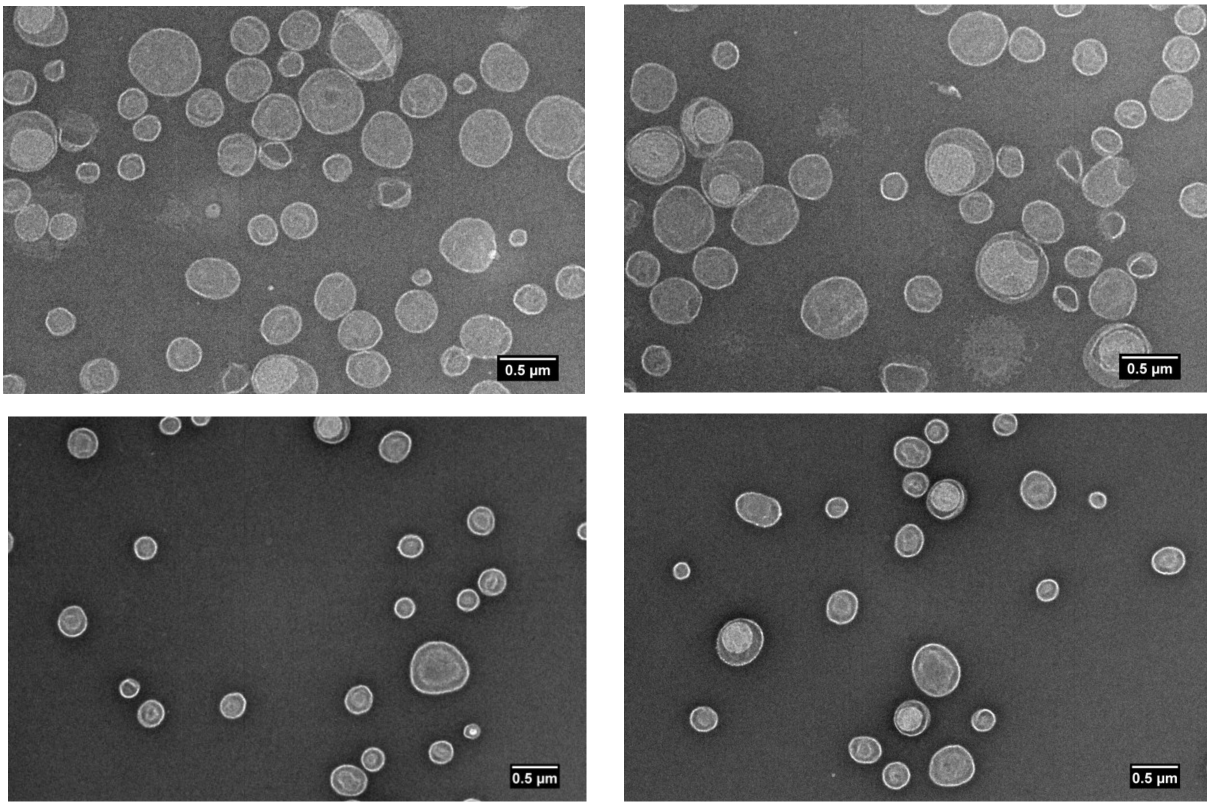

Figure S13. Representative TEM images of PEG_{113}-*b*-$HPMA_{300}$ formed at 15 wt% HPMA (ULV).

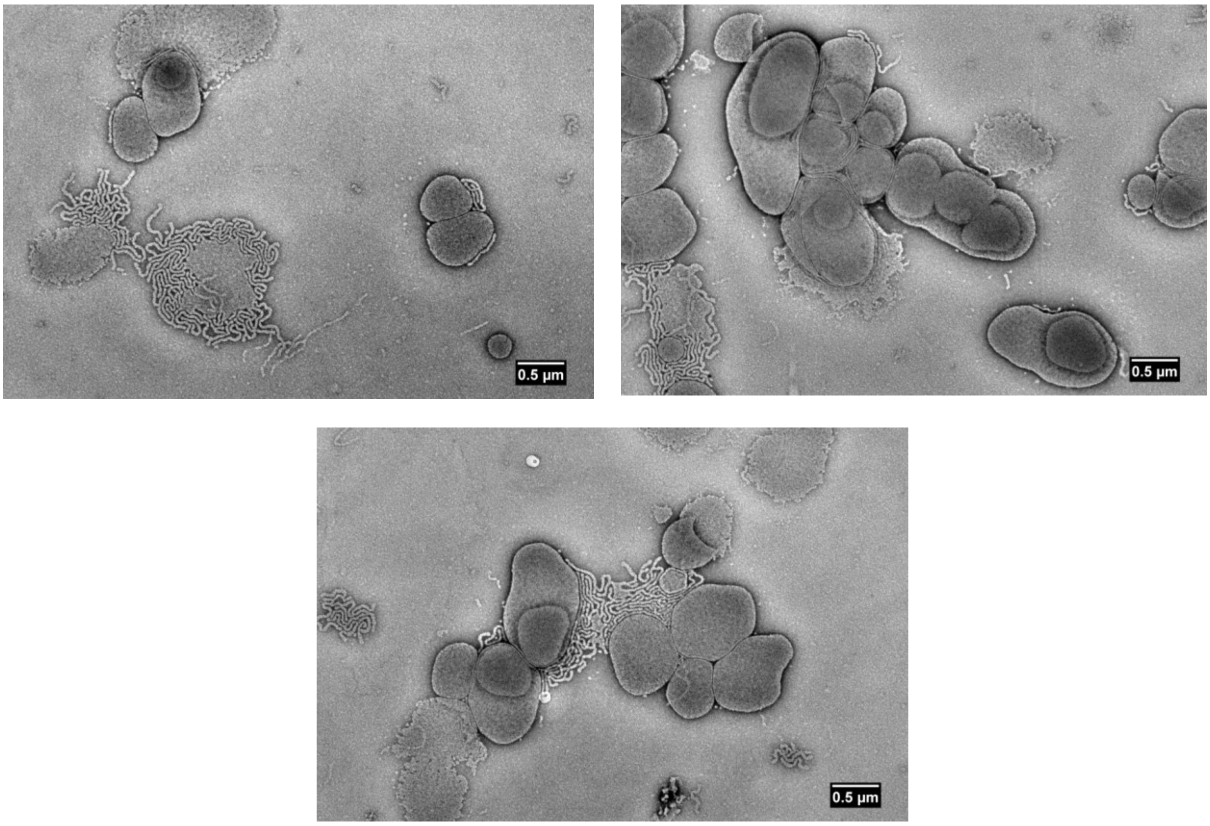

Figure S14. Representative TEM images of PEG_{113}-*b*-$HPMA_{300}$ formed at 20 wt% HPMA (W+ULV+MLV).

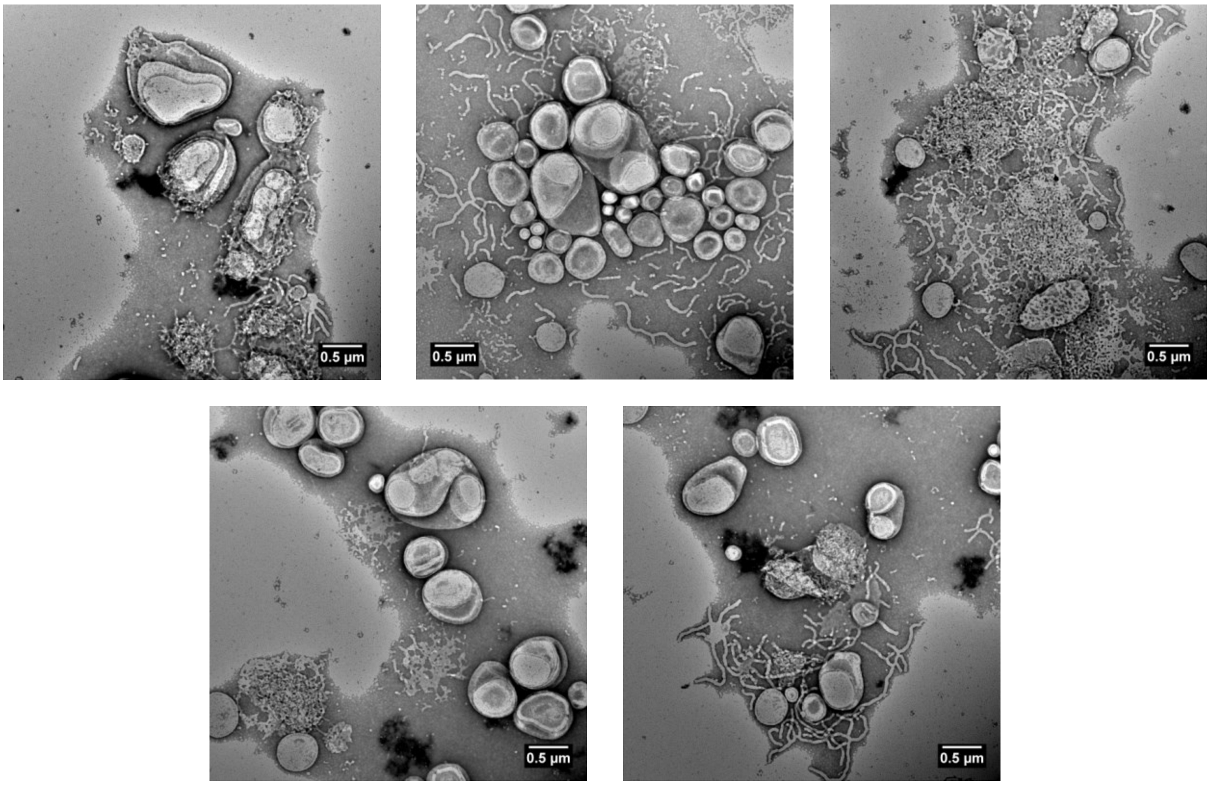

Figure S15. Representative TEM images of PEG_{113}-*b*-$HPMA_{300}$ formed at 25 wt% HPMA (W+ULV+MLV).

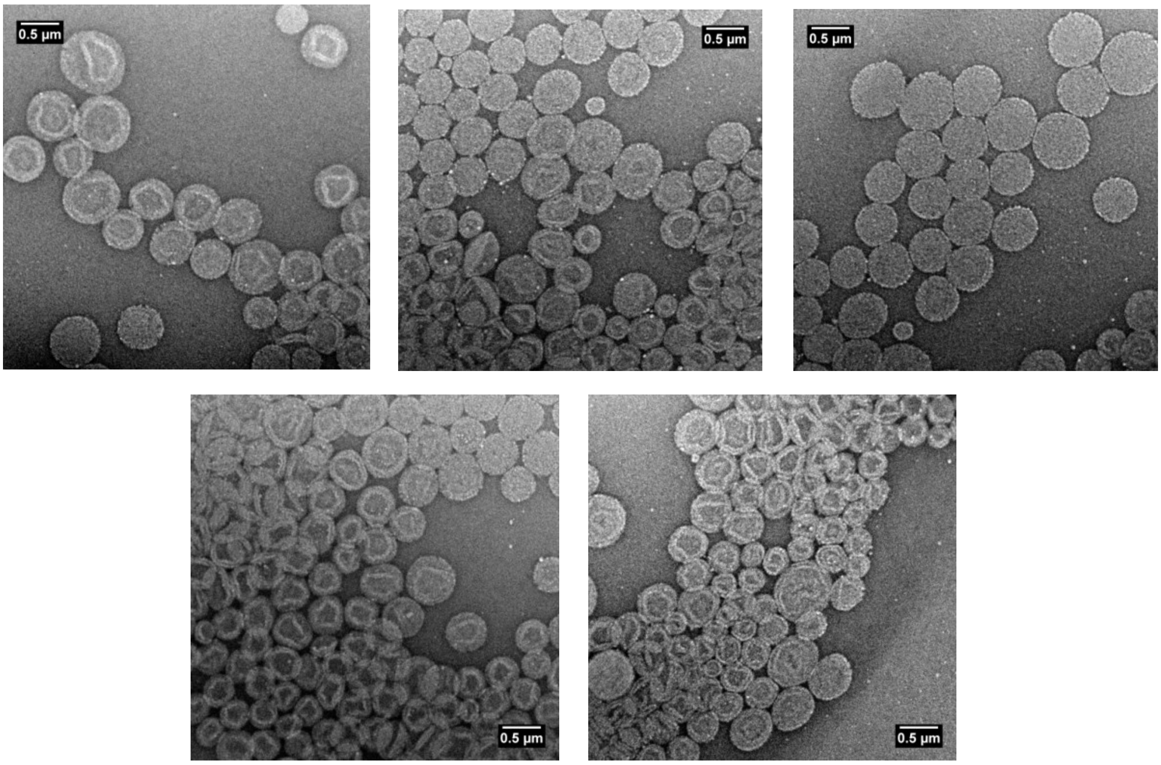

Figure S16. Representative TEM images of PEG_{113}-*b*-$HPMA_{400}$ formed at 10 wt% HPMA (ULV).

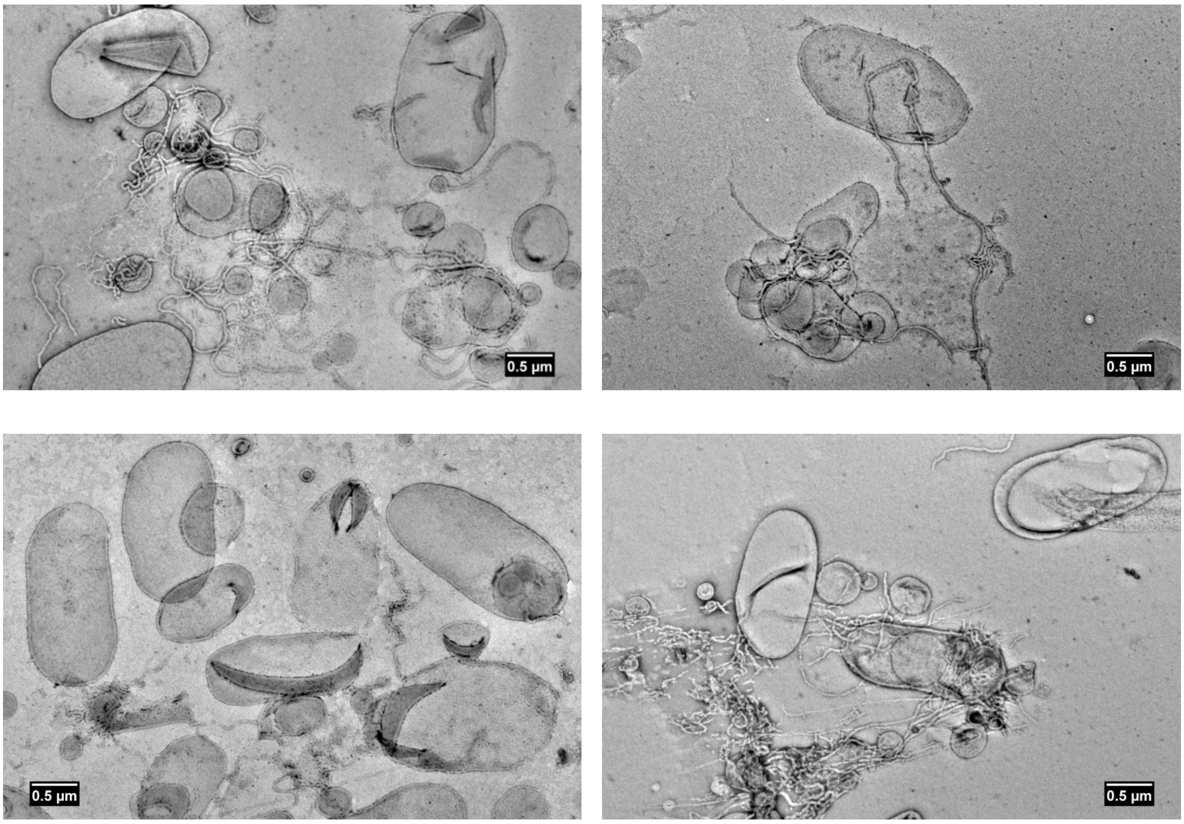

Figure S17. Representative TEM images of PEG_{113}-*b*-$HPMA_{400}$ formed at 15 wt% HPMA (W+ULV+MLV).

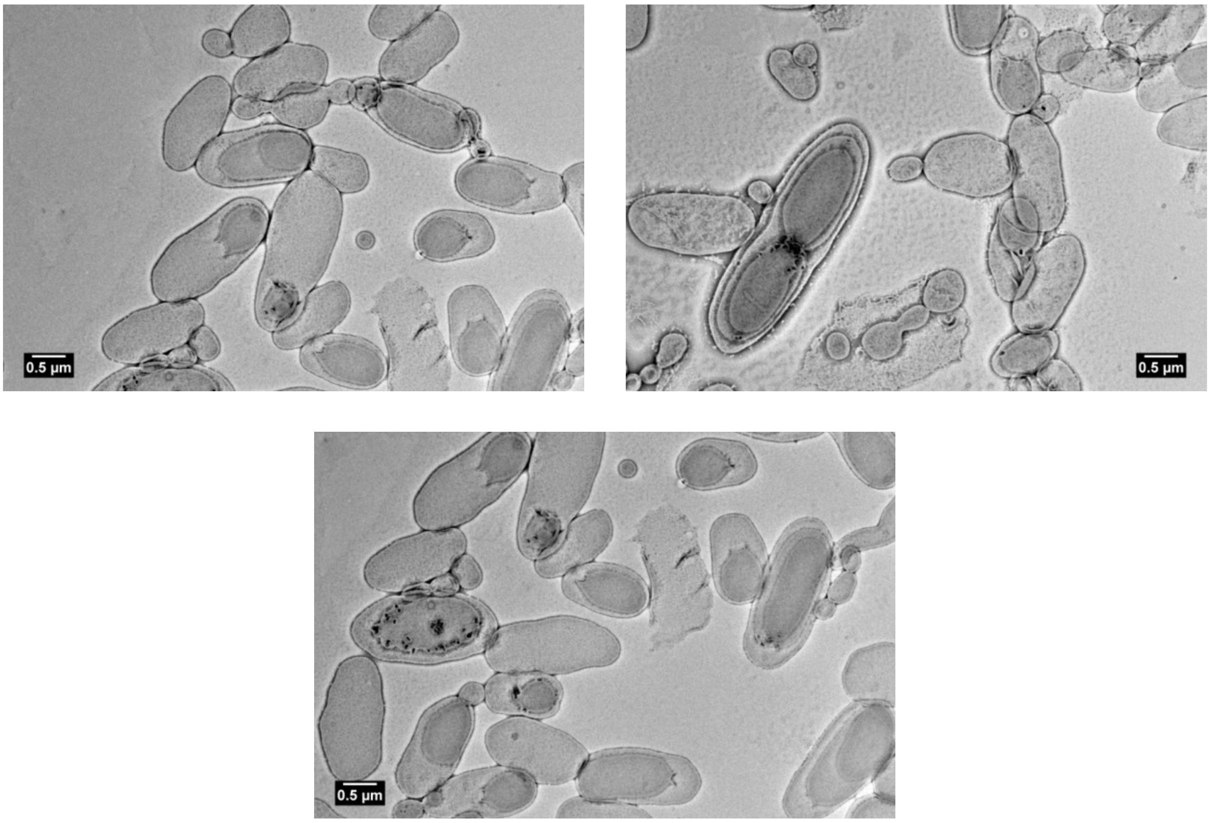

Figure S18. Representative TEM images of PEG_{113}-*b*-$HPMA_{400}$ formed at 20 wt% HPMA (ULV+MLV).

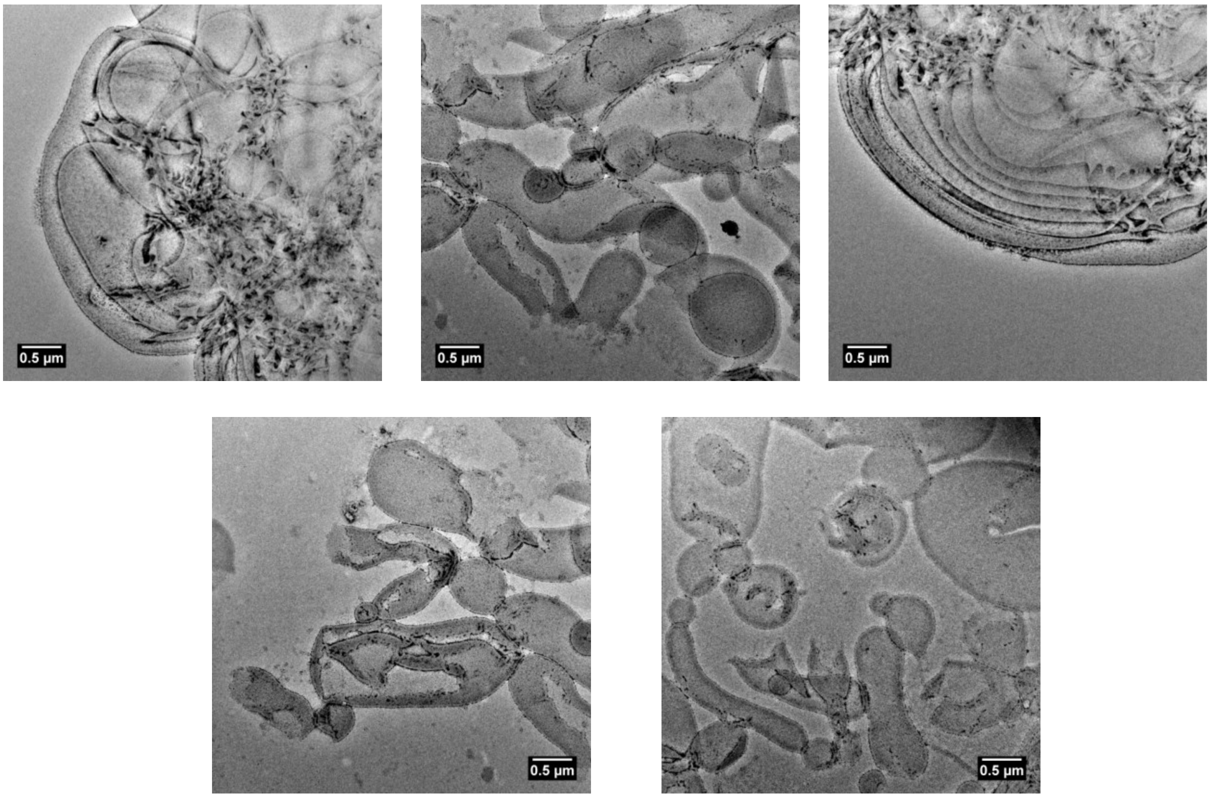

Figure S19. Representative TEM images of PEG_{113}-*b*-$HPMA_{400}$ formed at 25 wt% HPMA (ULV+MLV).

Supplementary TEM data for formulations formed by route B

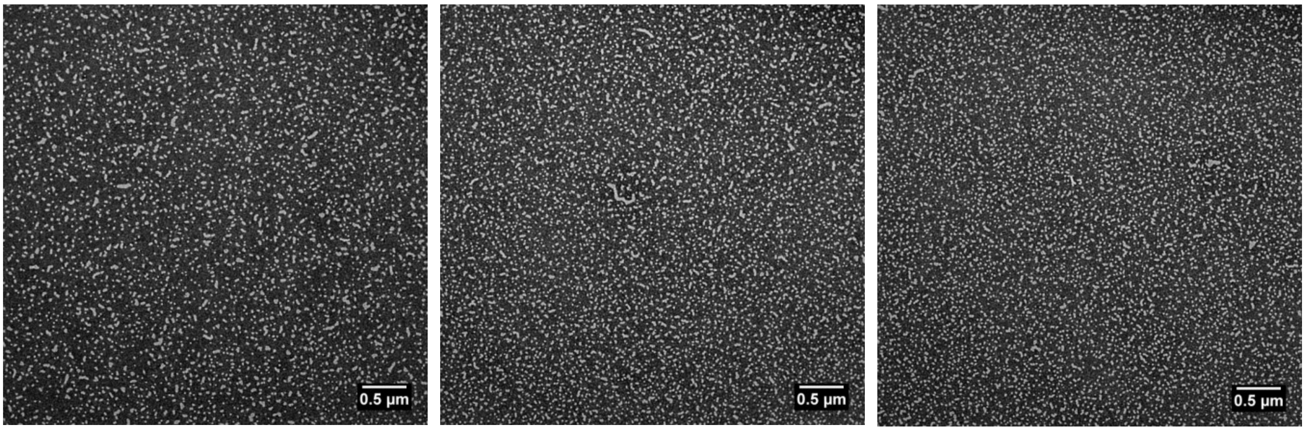

Figure S20. Representative TEM images of PEG_{113}-*b*-$HPMA_{100}$ formed at 10 wt% HPMA (S).

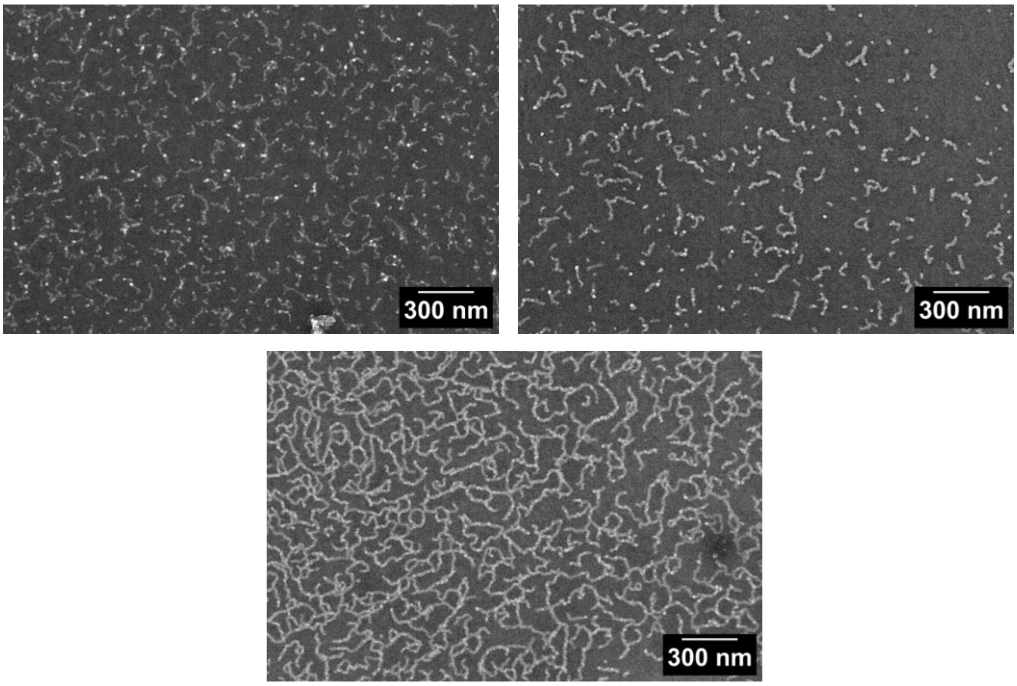

Figure S21. Representative TEM images of PEG_{113}-*b*-$HPMA_{100}$ formed at 15 wt% HPMA (S+W).

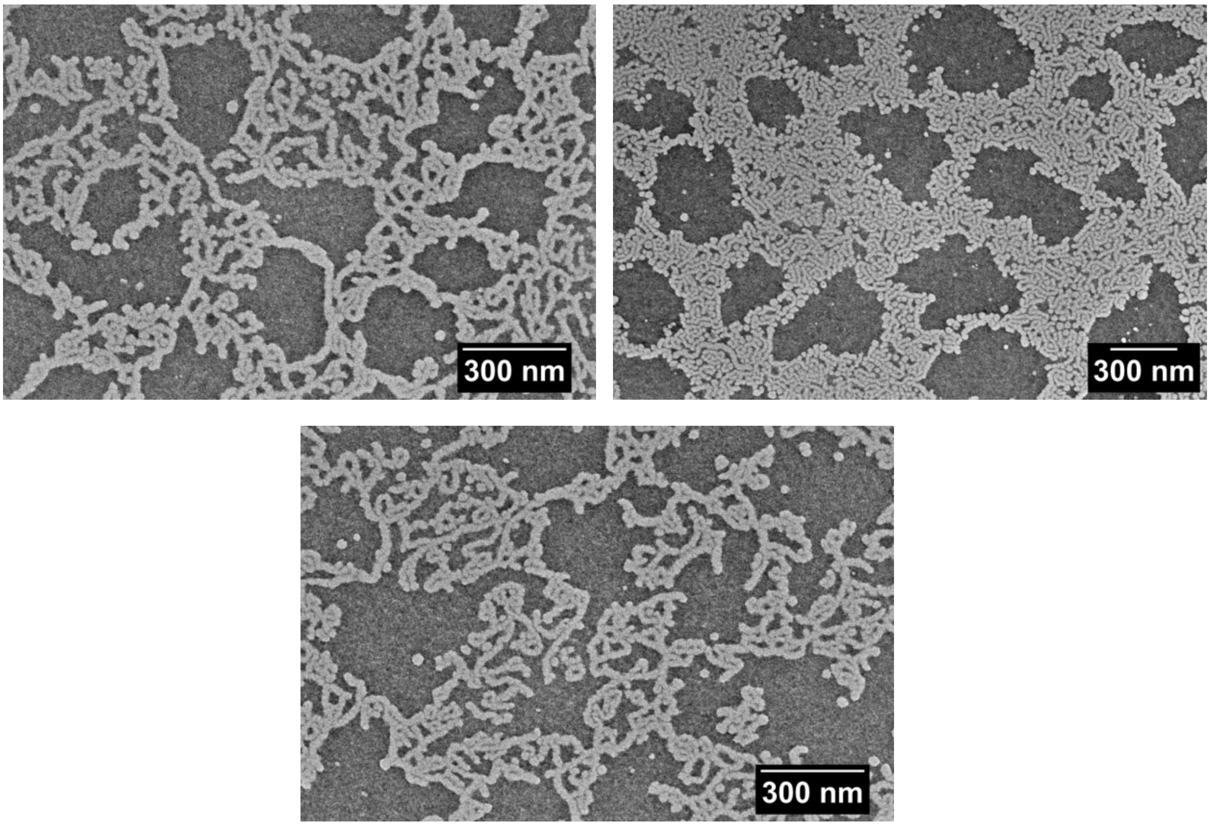

Figure S22. Representative TEM images of PEG_{113}-*b*-$HPMA_{100}$ formed at 20 wt% HPMA (S+W).

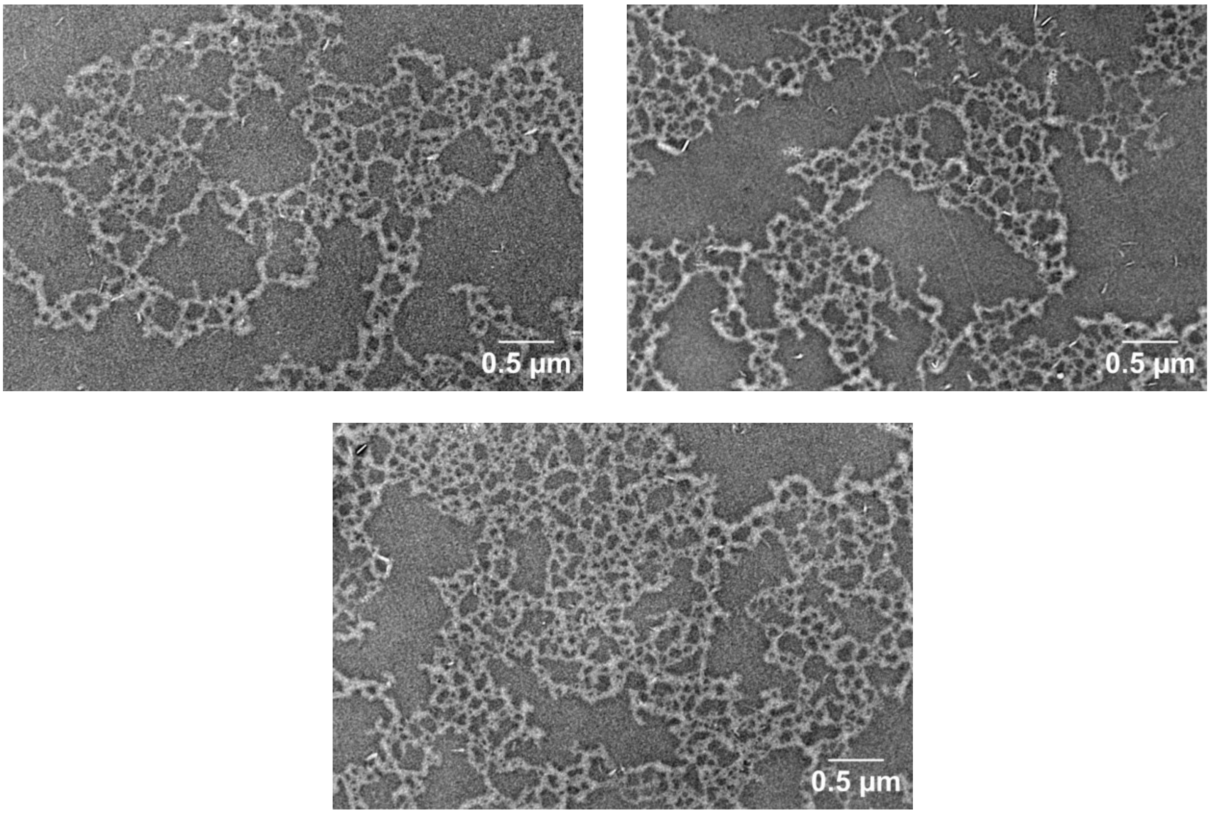

Figure S23. Representative TEM images of PEG_{113}-*b*-$HPMA_{100}$ formed at 25 wt% HPMA (W).

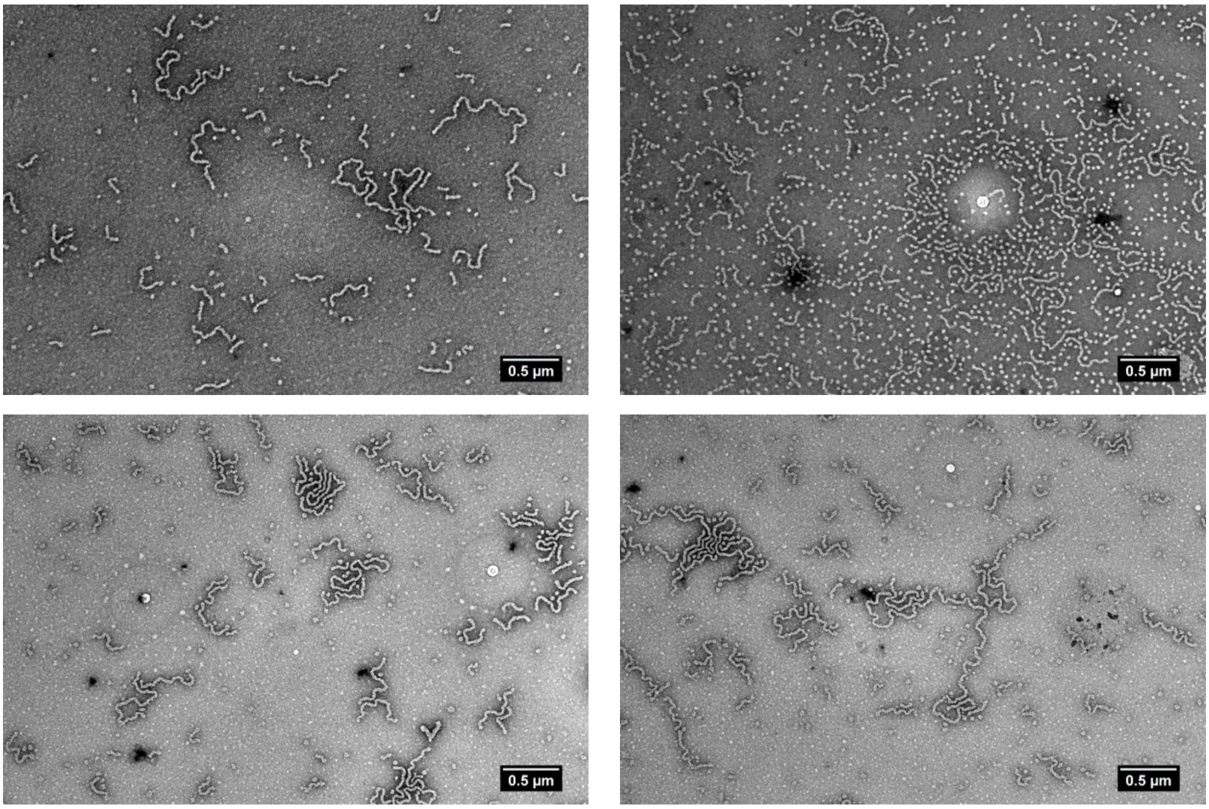

Figure S24. Representative TEM images of PEG_{113}-*b*-$HPMA_{200}$ formed at 10 wt% HPMA. (S+W).

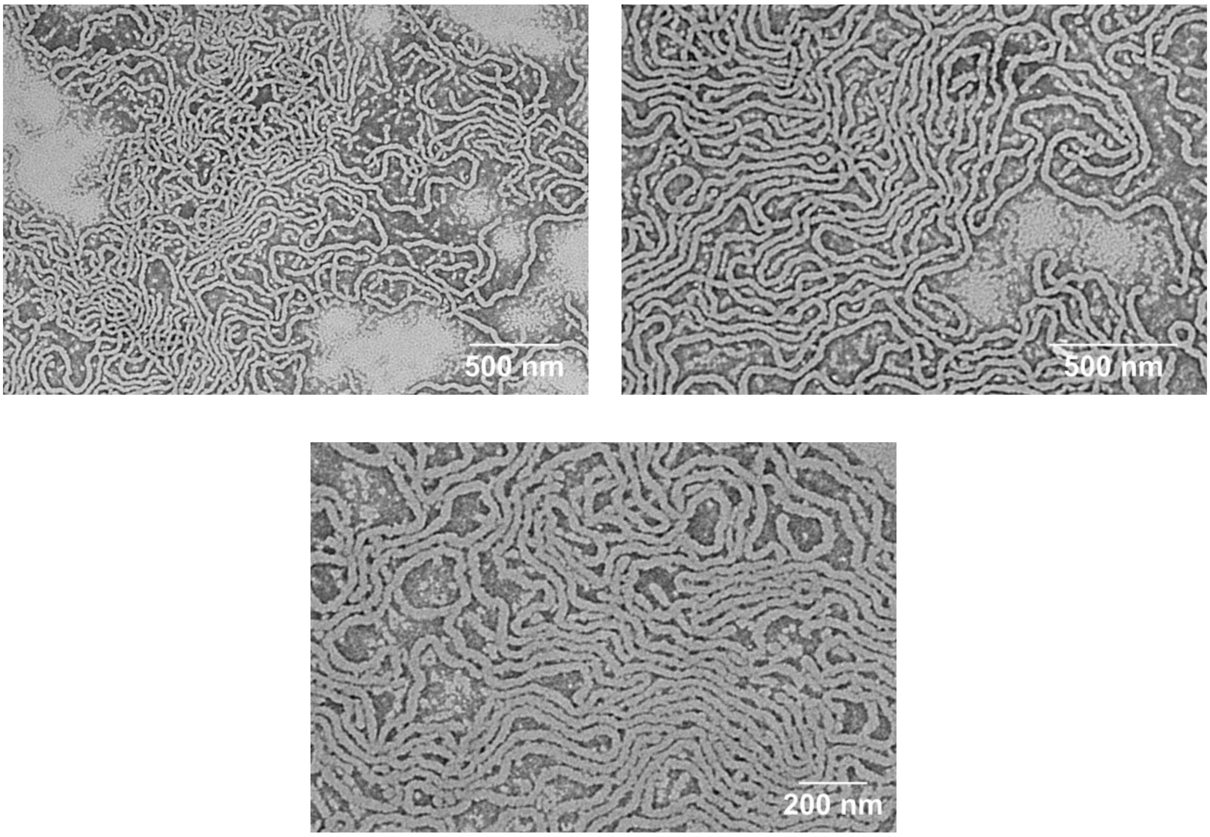

Figure S25. Representative TEM images of PEG_{113}-*b*-$HPMA_{200}$ formed at 15 wt% HPMA (W).

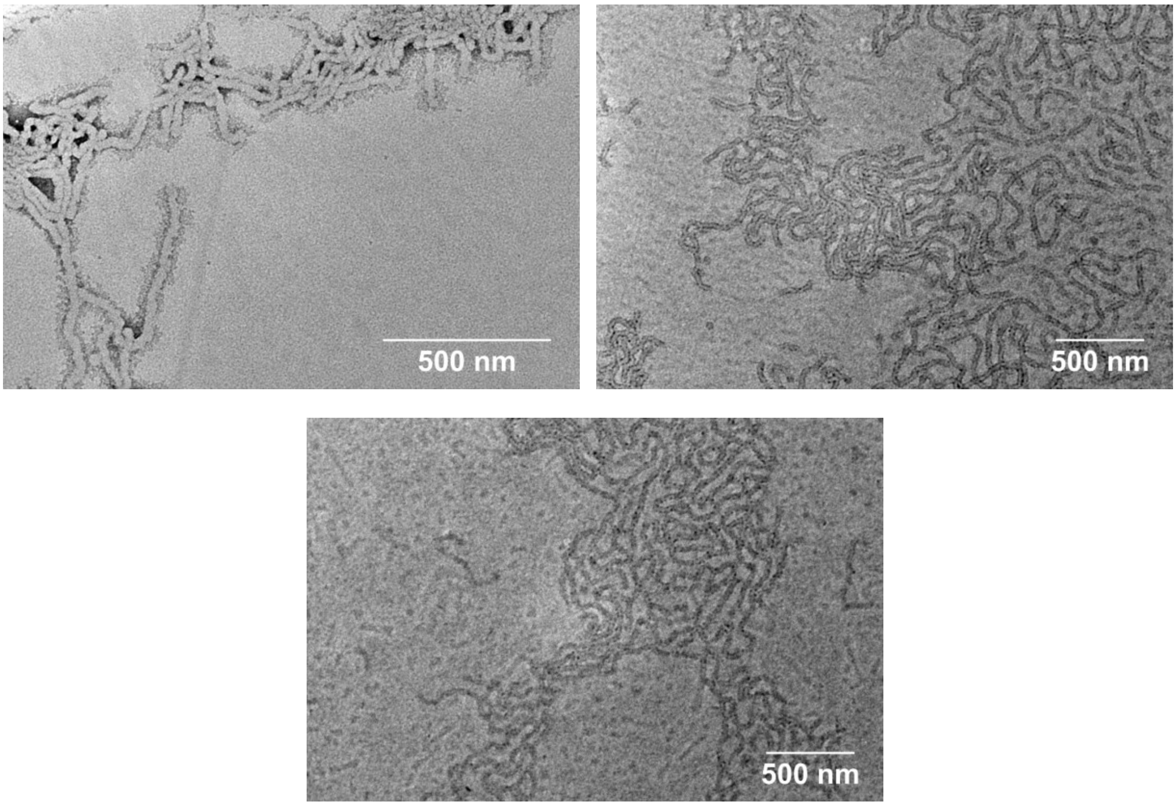

Figure S26. Representative TEM images of PEG_{113}-*b*-$HPMA_{200}$ formed at 20 wt% HPMA (W).

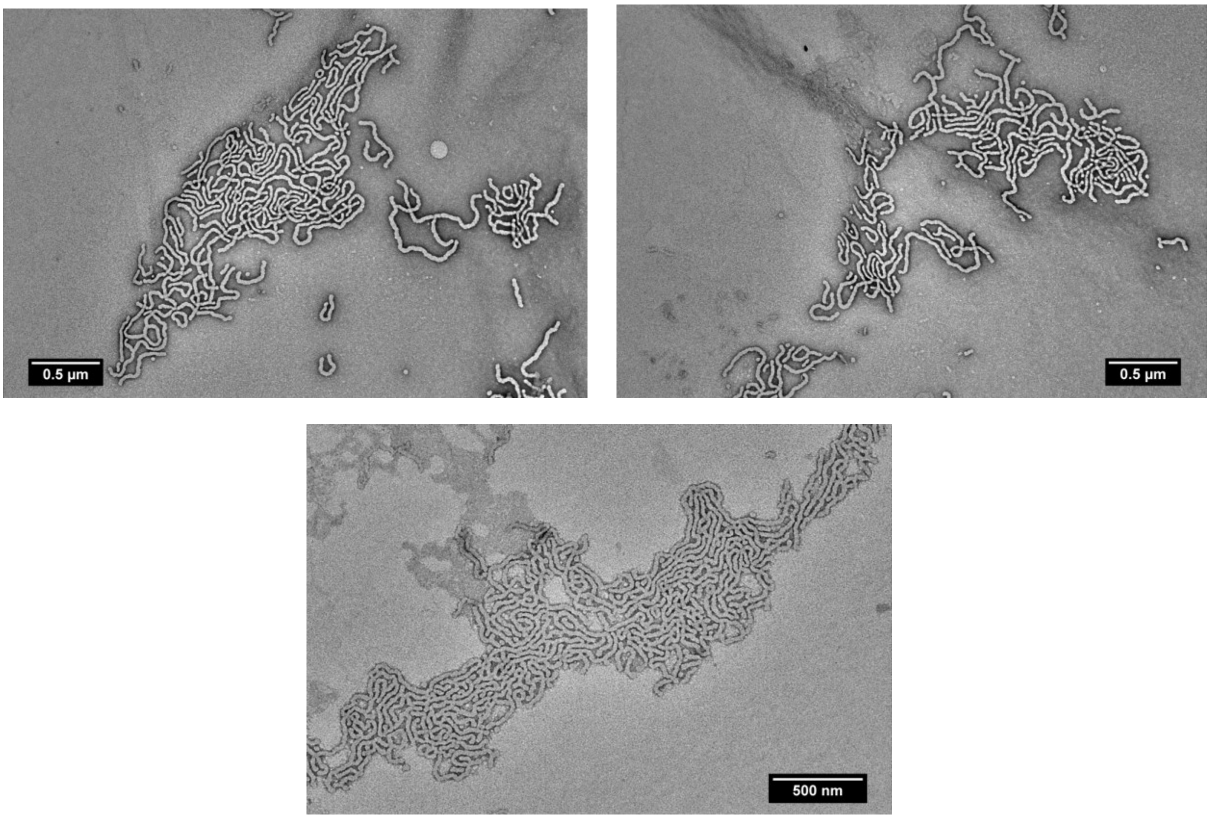

Figure S27. Representative TEM images of PEG_{113}-*b*-$HPMA_{200}$ formed at 25 wt% HPMA (W).

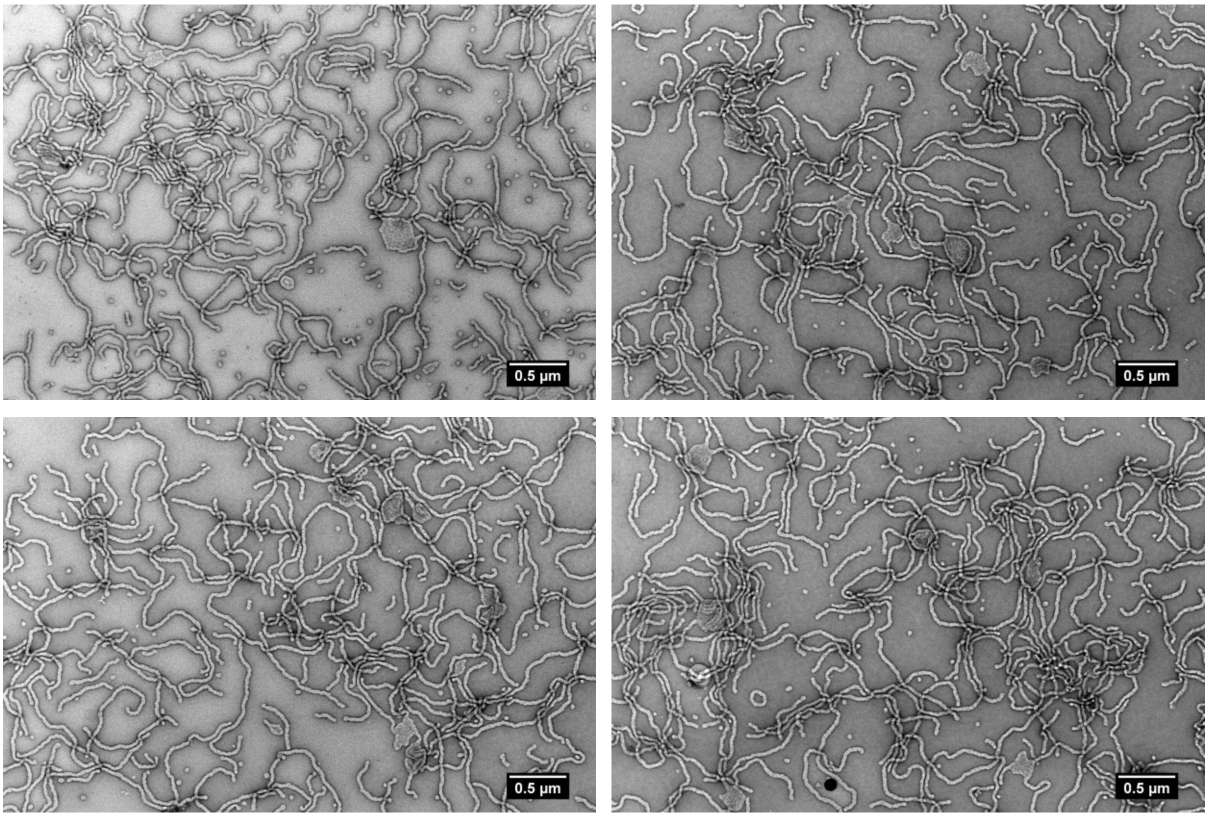

Figure S28. Representative TEM images of PEG_{113}-*b*-$HPMA_{300}$ formed at 10 wt% HPMA (S+W).

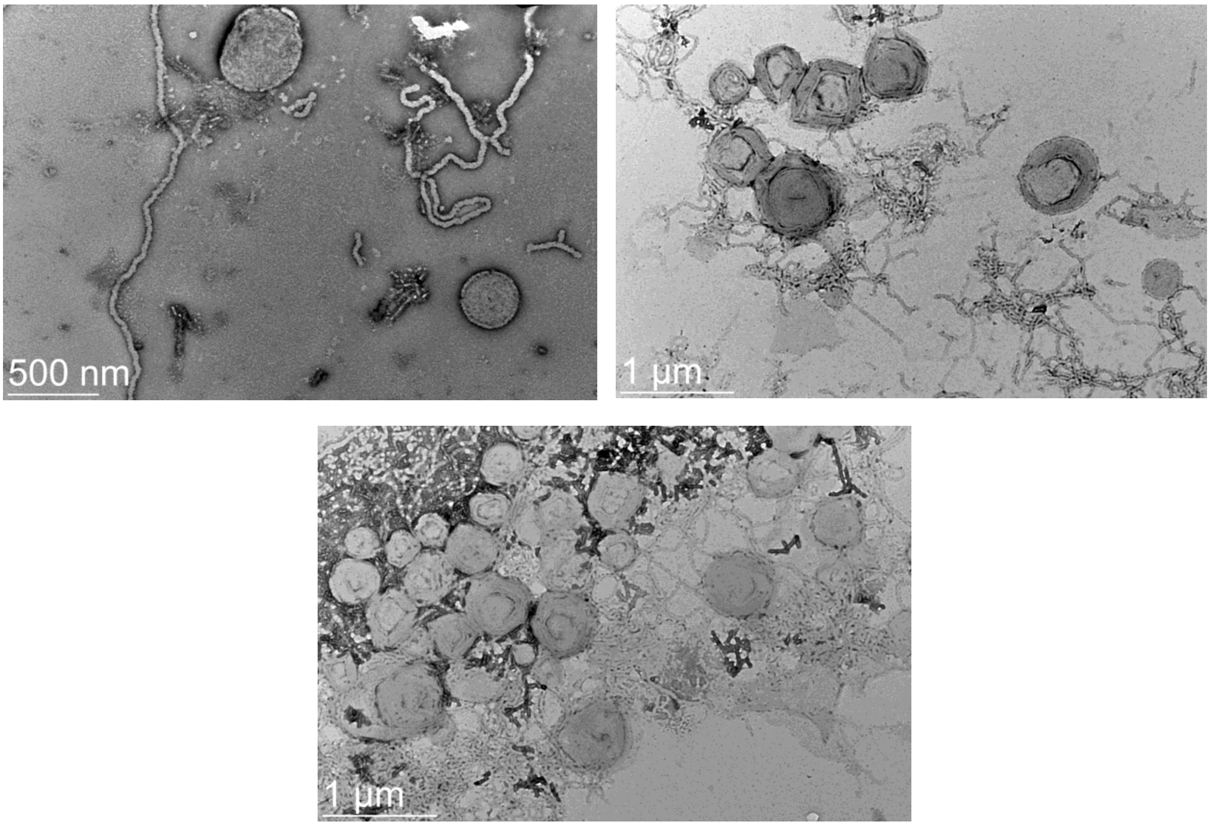

Figure S29. Representative TEM images of PEG_{113}-*b*-$HPMA_{300}$ formed at 15 wt% HPMA (W+ULV).

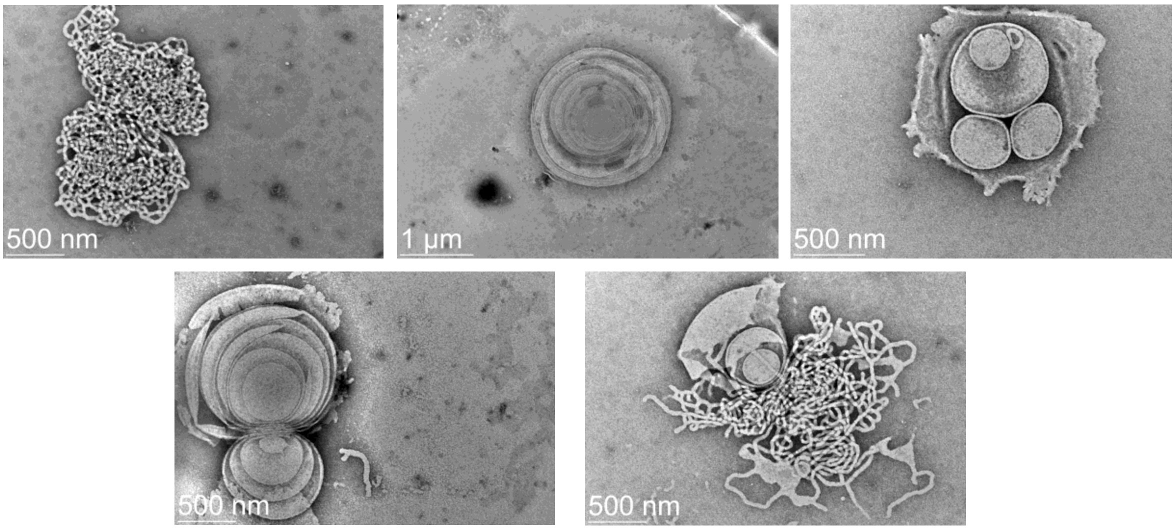

Figure S30. Representative TEM images of PEG_{113}-*b*-$HPMA_{300}$ formed at 20 wt% HPMA (W+ULV+MLV).

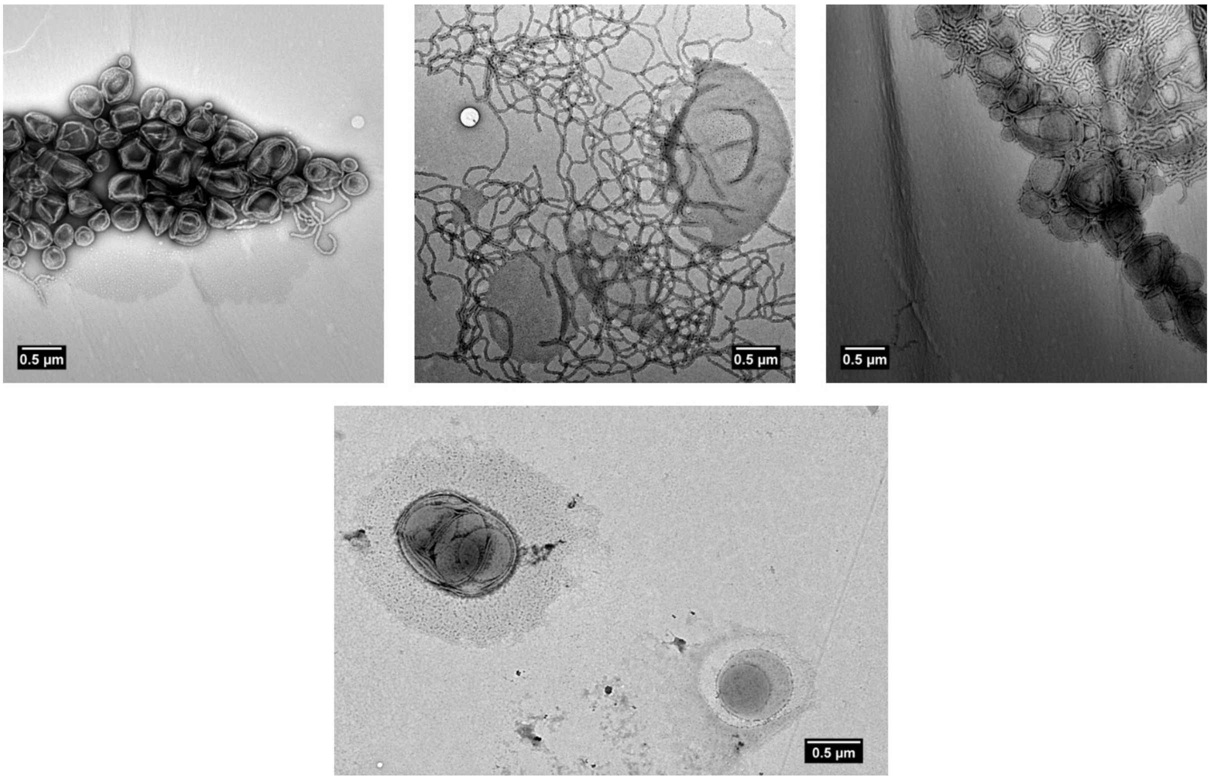

Figure S31. Representative TEM images of PEG_{113}-*b*-$HPMA_{300}$ formed at 25 wt% HPMA (W+ULV+MLV).

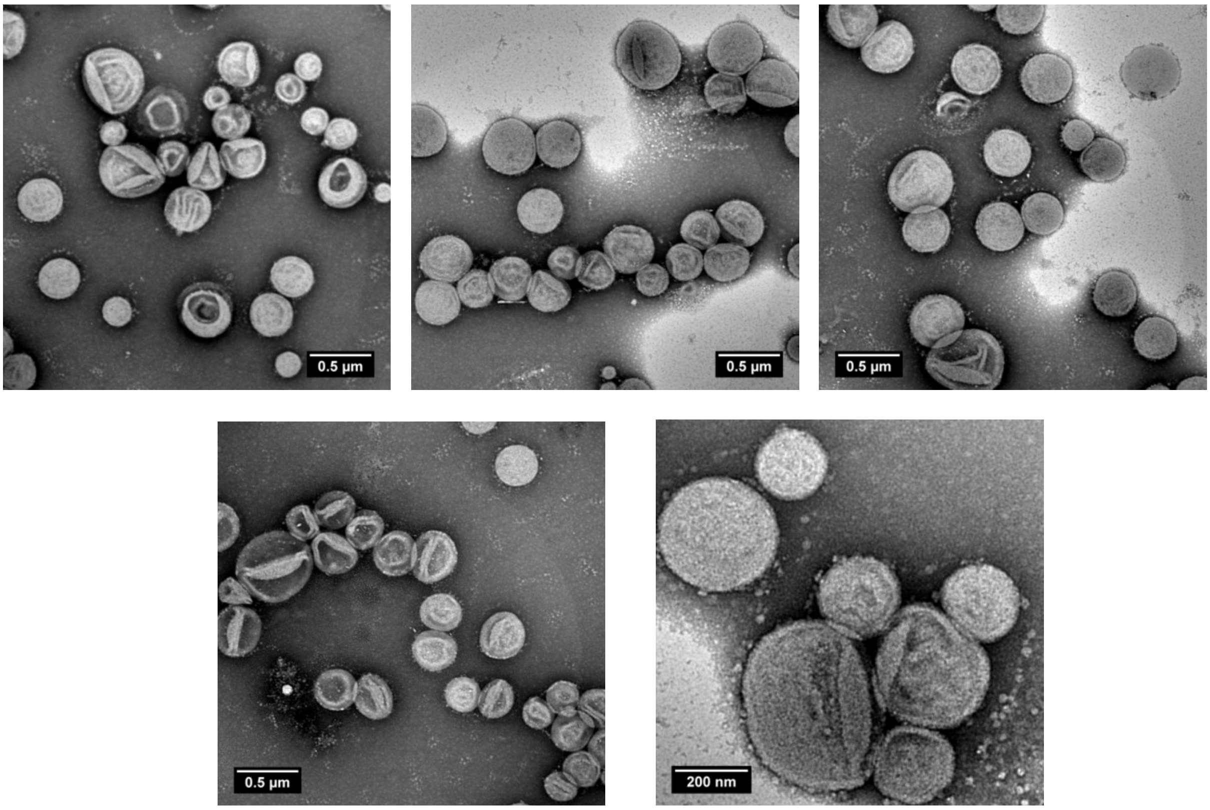

Figure S32. Representative TEM images of PEG$_{113}$-*b*-HPMA$_{400}$ formed at 10 wt% HPMA (ULV).

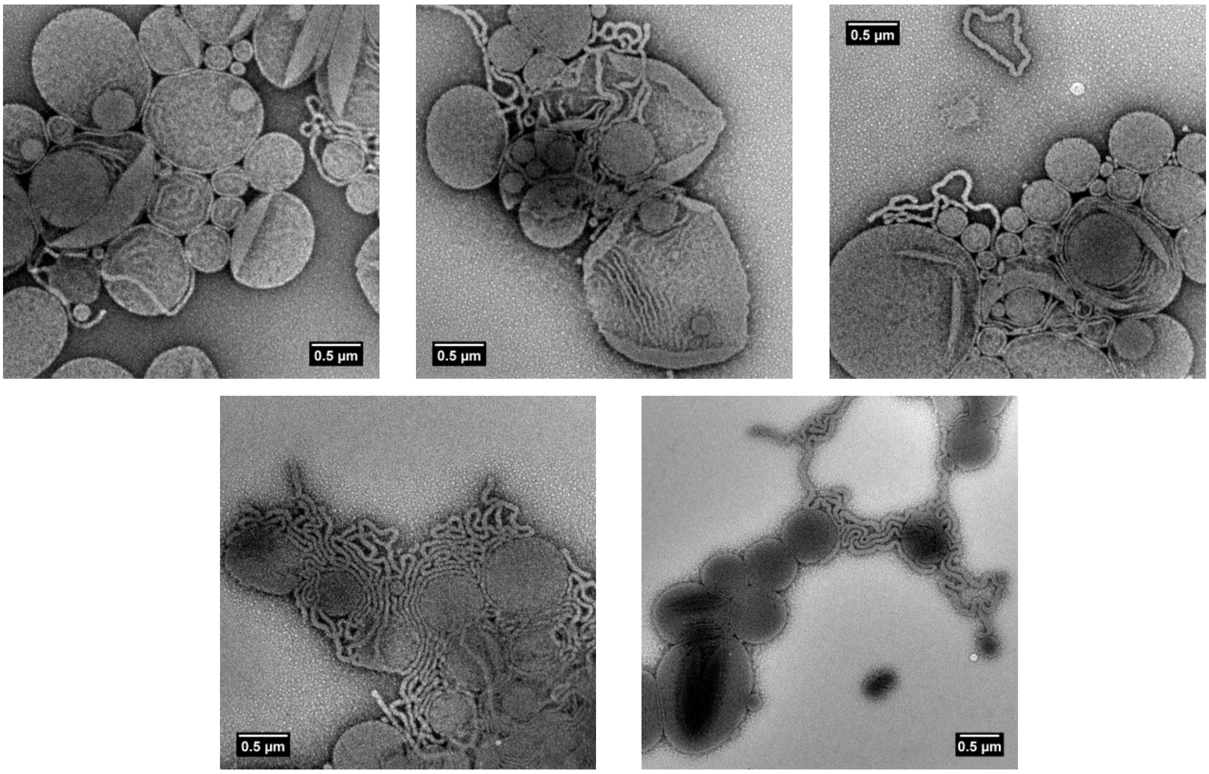

Figure S33. Representative TEM images of PEG$_{113}$-*b*-HPMA$_{400}$ formed at 15 wt% HPMA (W+ULV+MLV).

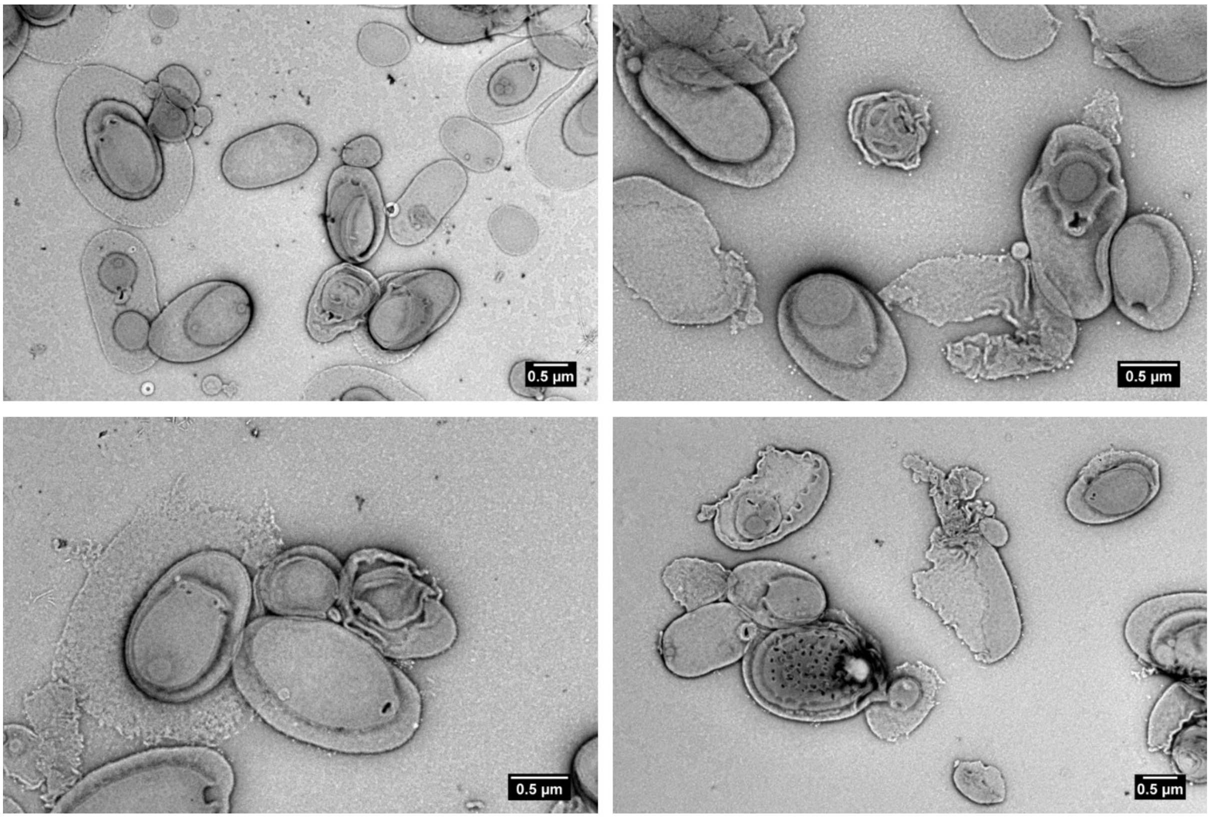

Figure S34. Representative TEM images of PEG_{113}-*b*-$HPMA_{400}$ formed at 20 wt% HPMA (ULV+MLV).

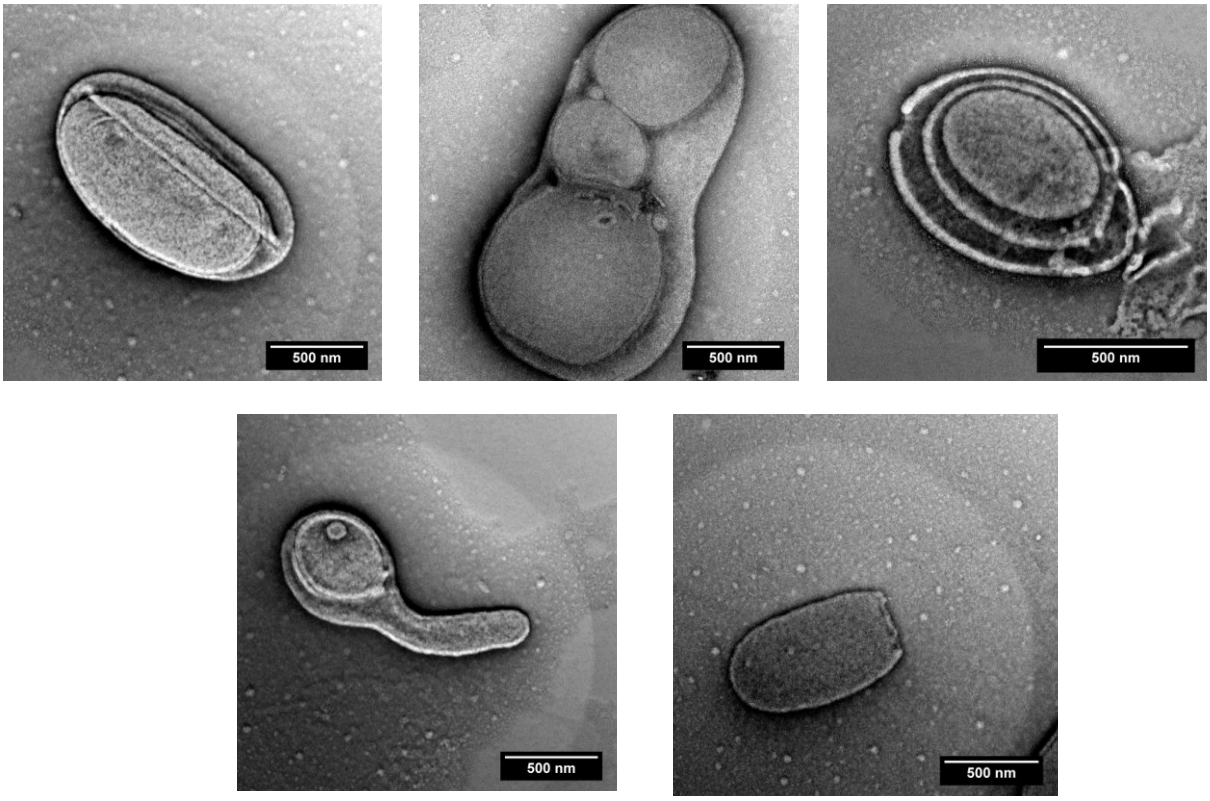

Figure S35. Representative TEM images of PEG_{113}-*b*-$HPMA_{400}$ formed at 25 wt% HPMA (ULV+MLV).

Supplementary DLS data for formulations formed by routes A and B

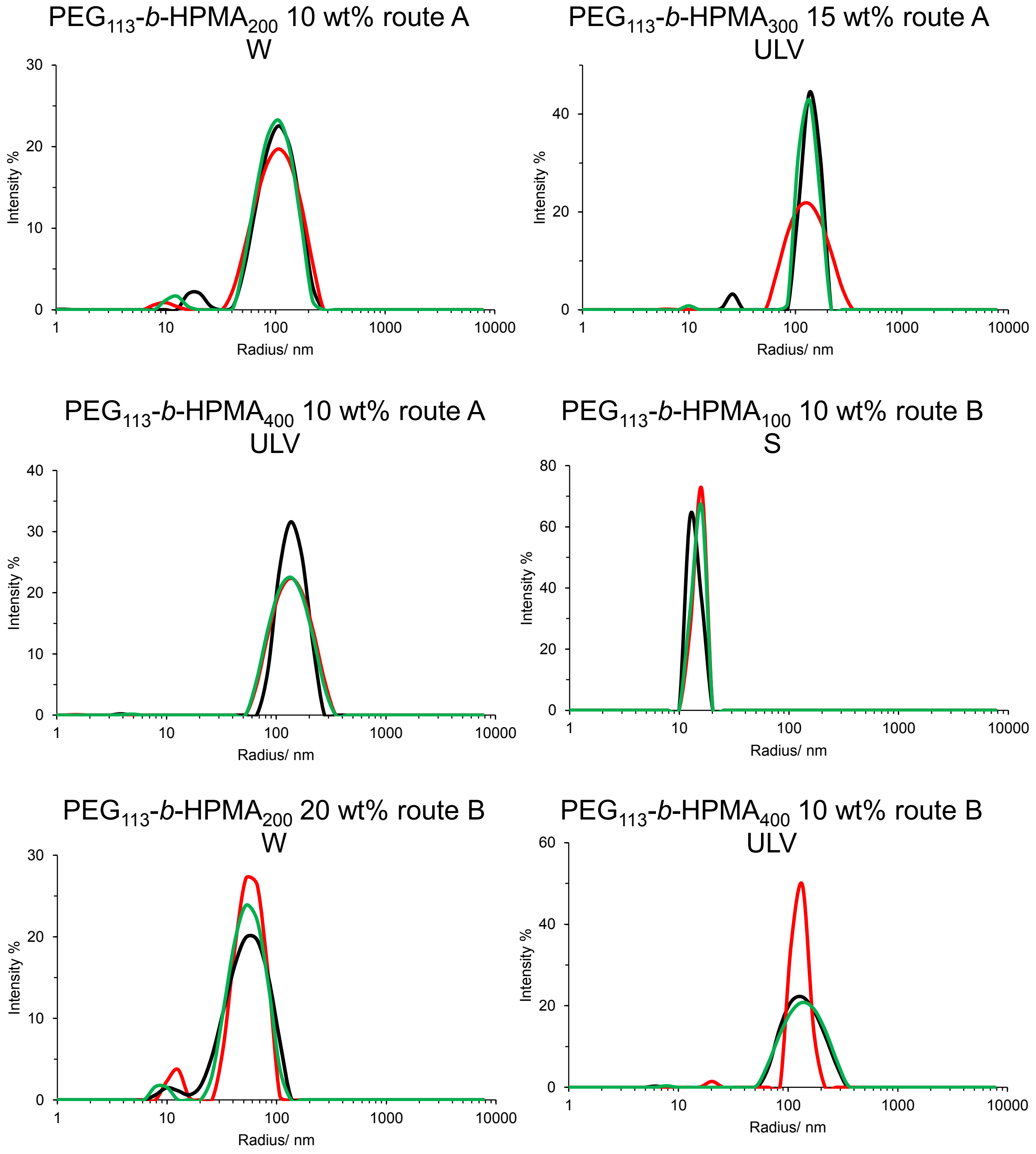

Figure S36. Intensity weighted size distributions of some representative pure phases obtained by DLS of PEG$_{113}$-*b*-HPMA$_x$ formulations obtained by routes A or B at various concentrations, as indicated above each graph. Black, red and green traces represent separate repeat measurements. The morphology obtained from TEM is also stated above for reference.

Histograms of PEG_{113}-*b*-$HPMA_{400}$ unilamellar vesicles

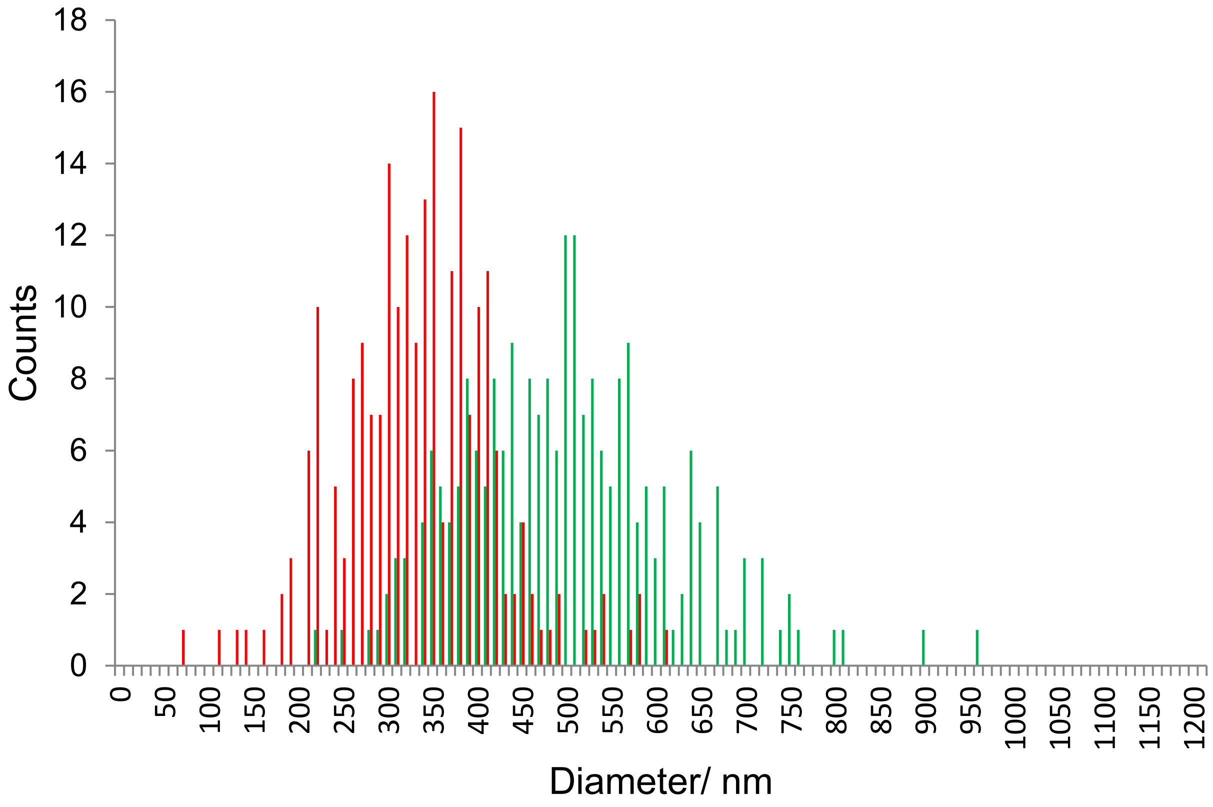

Fig. S37. Histograms of PEG_{113}-*b*-$PHPMA_{400}$ diblock copolymer unilamellar vesicles formed at [HPMA] = 10 wt% by route A (green) and route B (red) measured from particle counting measurements on TEM images. In each case at least 200 particles were analyzed.

Supplementary data for formulations formed by route C

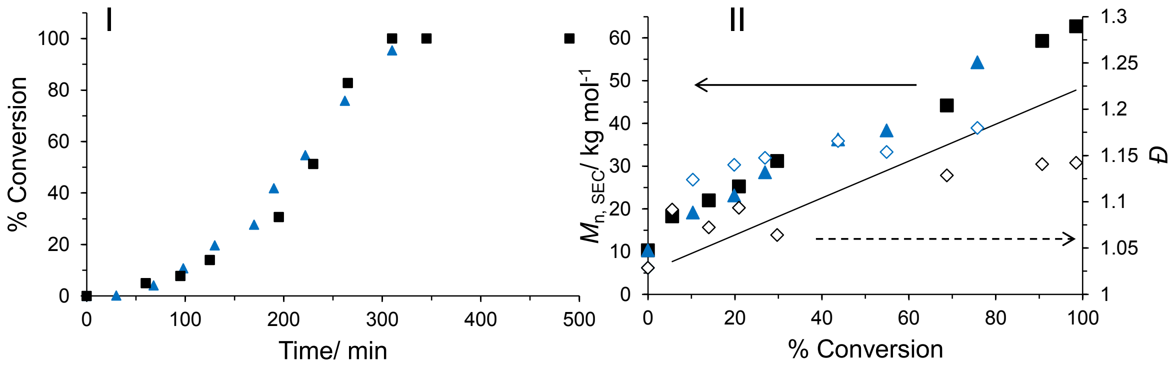

Figure S38. I: Kinetic study of the PEG_{113}-*b*-$HPMA_{300}$ formulation at 10 wt% formed by route C (blue triangles) overlaid with the kinetic data previously obtained for that formed by route B (black squares). II: SEC data for the kinetic studies. Left axis: M_n values of polymers formed by route C (blue triangles) and route B (black squares), the black linear trend shows the expected M_n values. Right axis: $Đ$ values of polymers formed by route C (blue triangles) and route B (black diamonds).

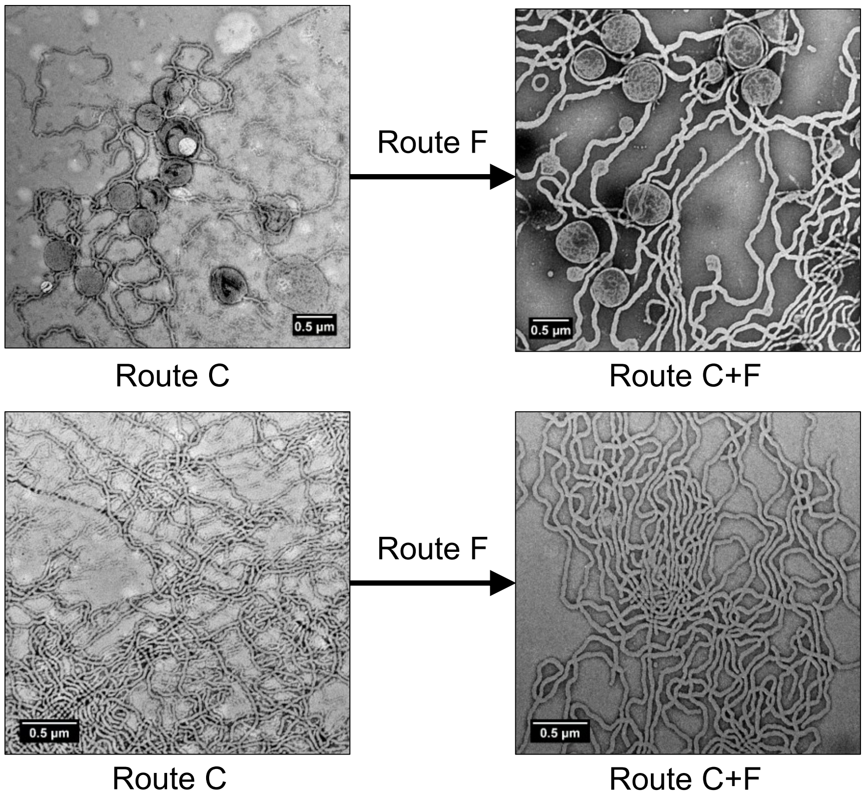

Figure S39. Representative TEM images showing no morphology change in formulations formed by route C (photoinitiation 20% light intensity at 37 °C) post-synthetically treated with route F (irradiation with 20% light intensity at 37 °C for 18 h). PEG_{113}-*b*-$HPMA_{300}$ at 10 wt% HPMA (top) and PEG_{113}-*b*-$HPMA_{200}$ at 15 wt% HPMA (bottom) are shown.

Supplementary data for the post-synthetic irradiation experiments

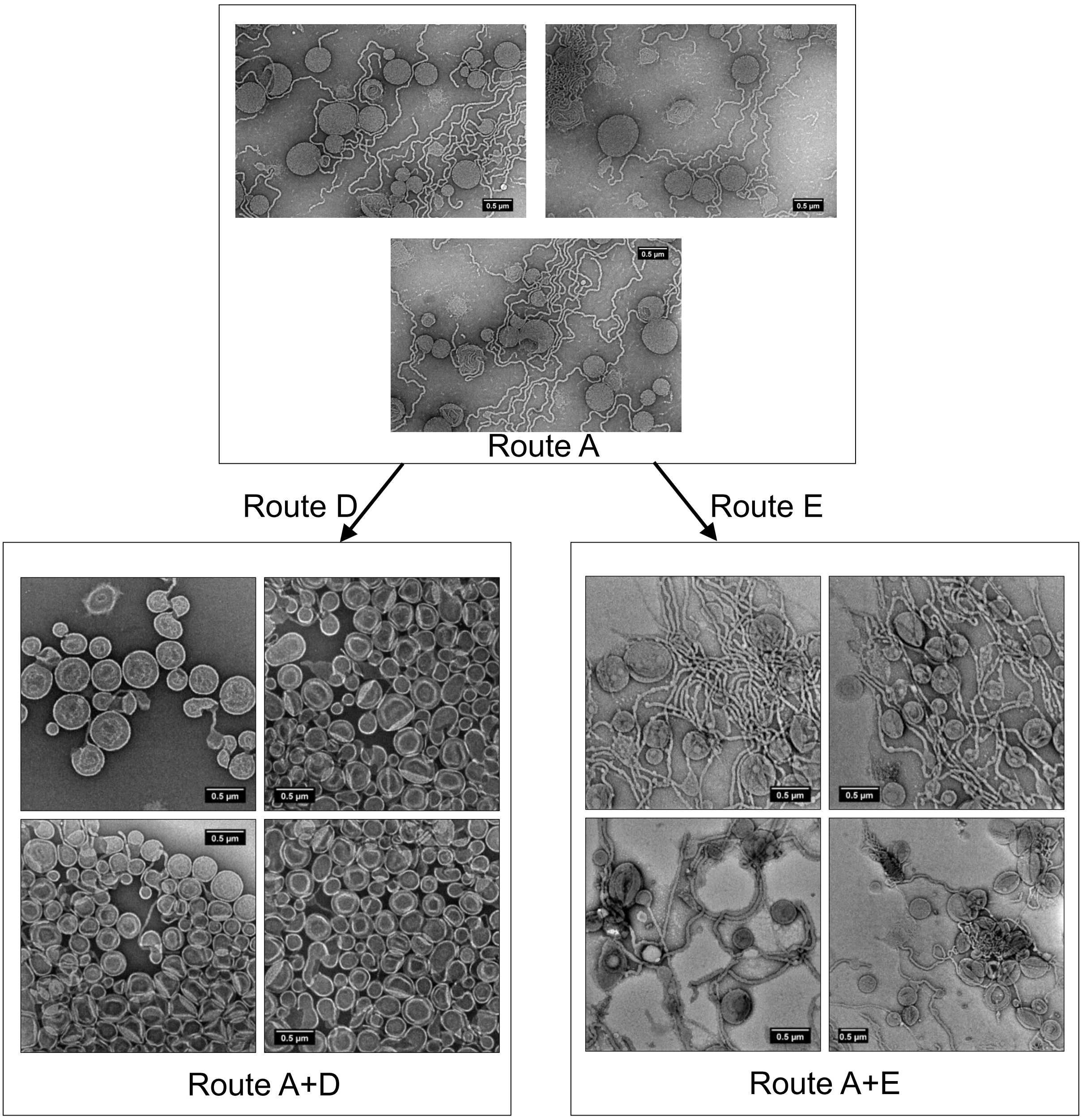

Figure S40. Representative TEM images showing morphology change of PEG$_{113}$-*b*-HPMA$_{300}$ at 10 wt% obtained by route A+D and A+E. Key: Route A = 100% light intensity photoinitiation at 37 °C. Route D = 100% light intensity photoirradiation at 37 °C for 18 h. Route E = incubation at 37 °C for 18 h.

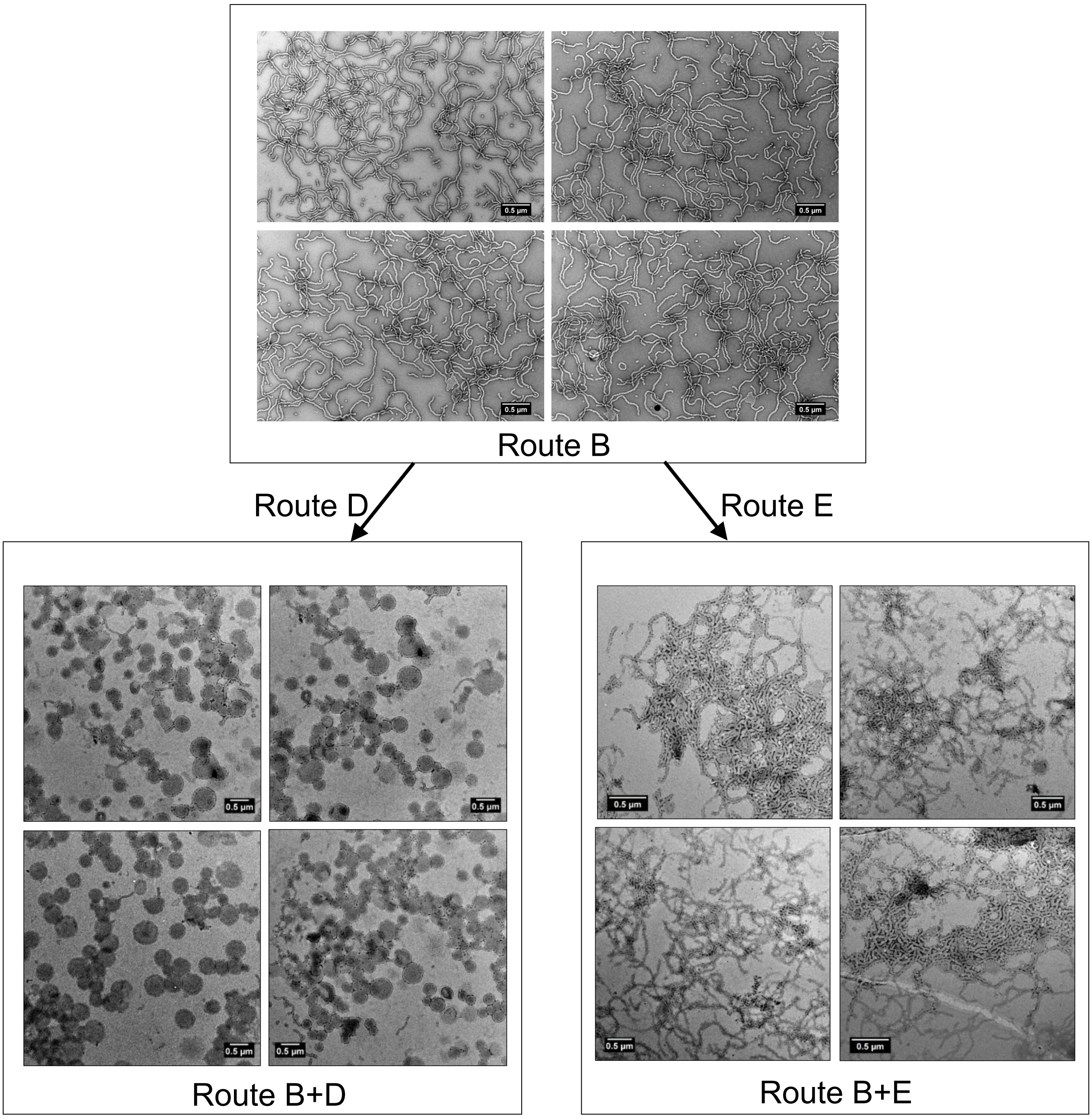

Figure S41. Representative TEM images showing morphology change of PEG_{113}-*b*-$HPMA_{300}$ at 10 wt% obtained by B+D and B+E. Key: Route A = Thermal initiation at 37 °C. Route D = 100% light intensity photoirradiation at 37 °C for 18 h. Route E = incubation at 37 °C for 18 h.

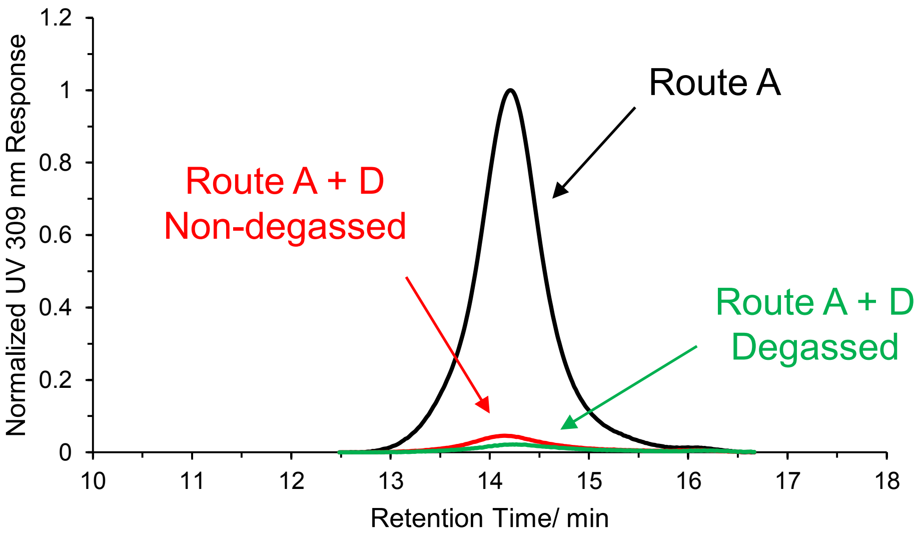

Figure S42. Normalized SEC UV traces of PEG_{113}-*b*-$HPMA_{400}$ formed at 10 wt% HPMA by route A (black) compared with those further irradiated with 100% light intensity for 18 h at 37 °C (route A+D) either with (green) or without (red) degassing the solution prior to irradiation.

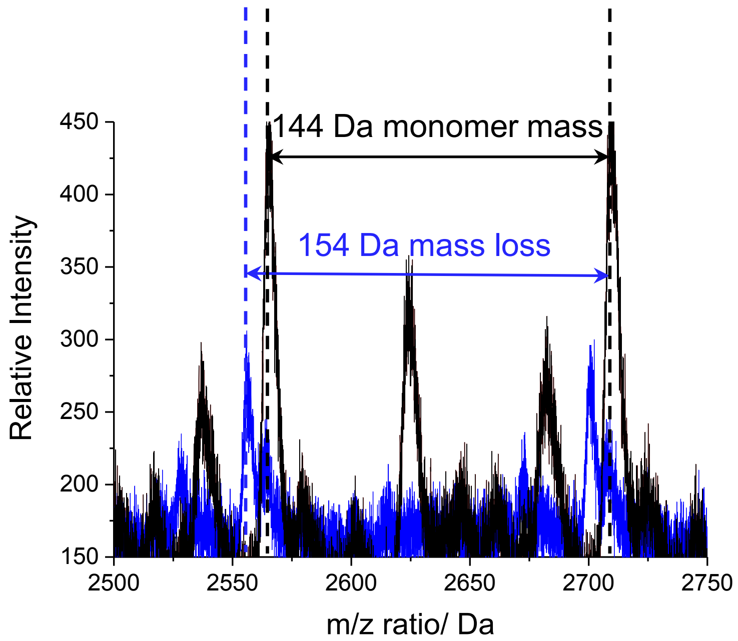

Figure S43. MALDI-ToF MS analysis of PHPMA oligomers before (black) and after (blue) irradiation with heat and light at 100% light intensity for 18 h.

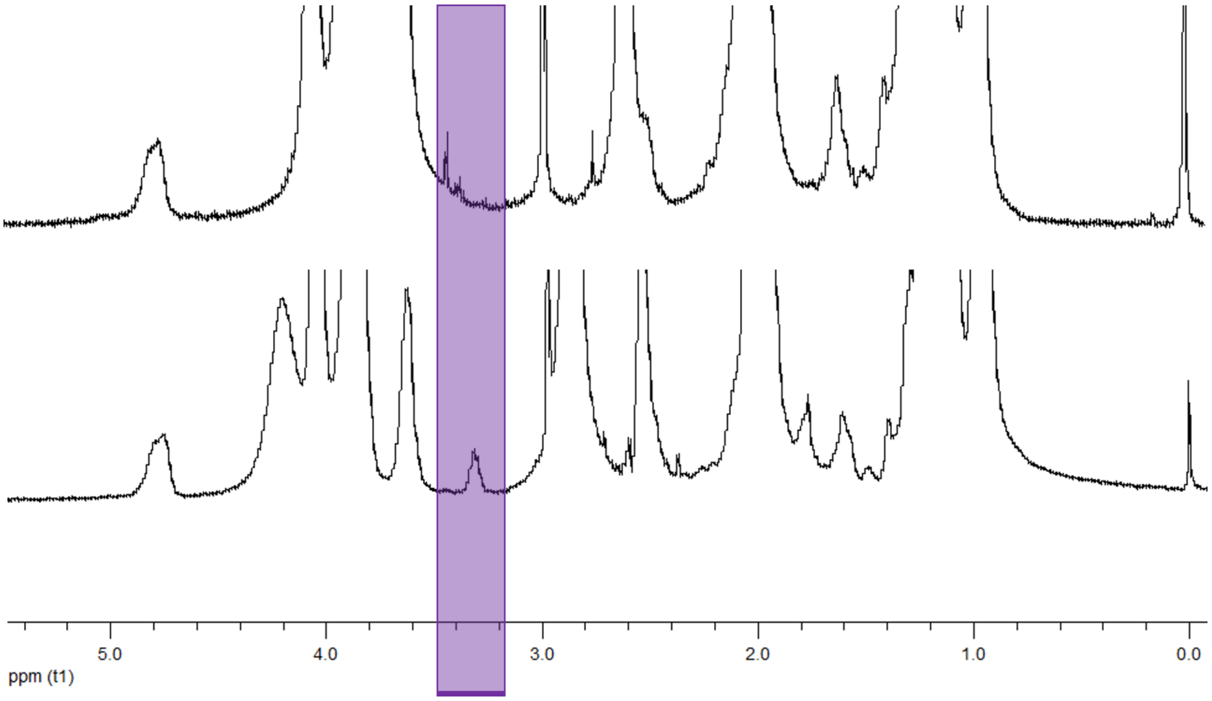

Figure S44. ^{1}H NMR spectroscopic analysis of PHPMA oligomers at 400 MHz in acetone-d6 before (bottom) and after (top) irradiation with heat and light at 100% light intensity for 18 h. Removal of the ethylene protons adjacent to the trithiocarbonate end group at 3.31 ppm after irradiation has been highlighted.

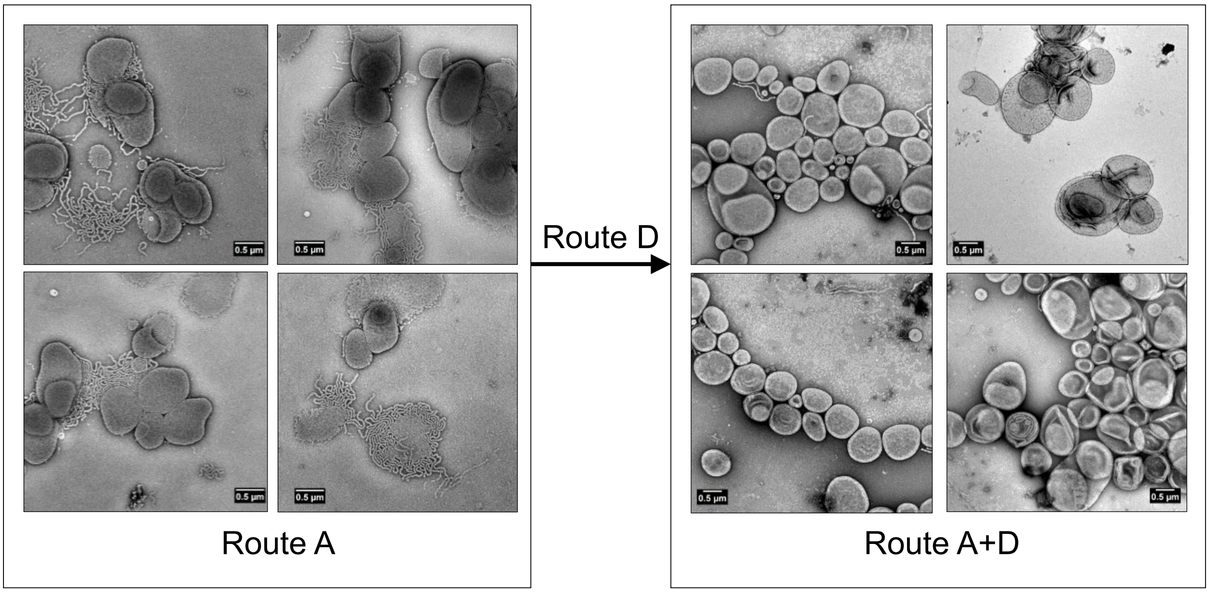

Figure S45. Representative TEM images showing morphology change of PEG_{113}-*b*-$HPMA_{300}$ at 20 wt% obtained by route A and route A+D. Key: Route A = 100% light intensity photoinitiation at 37 °C. Route D = 100% light intensity photoirradiation at 37 °C for 18 h.

Equilibrium structure of PEG$_{113}$-*b*-HPMA$_{300}$ formed by route A+D.

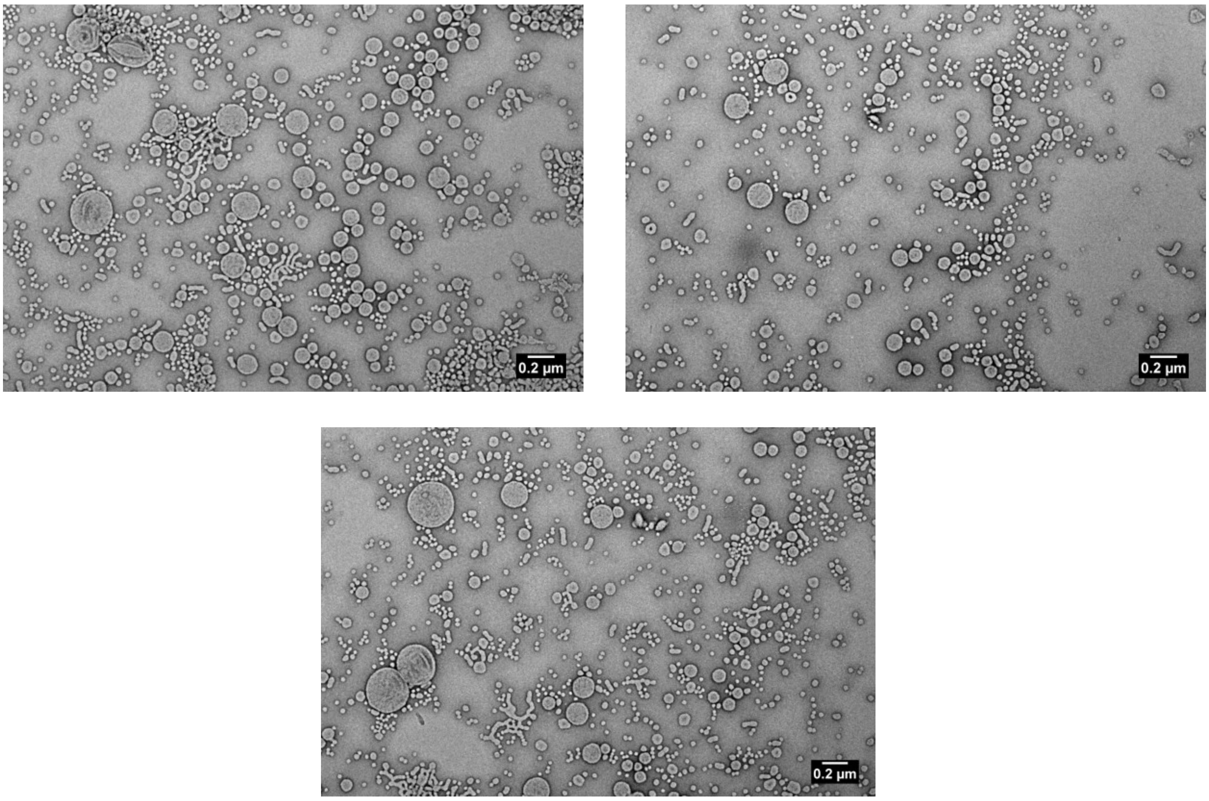

Figure S46. Representative TEM images of the new equilibrium structure at 1 mg mL^{-1} of PEG$_{113}$-*b*-HPMA$_{300}$ obtained by route A+D at 10 wt%.
